# Emergent collective dynamics of microrobotic swarms in viscoelastic media: a computational perspective

**DOI:** 10.1140/epje/s10189-026-00617-4

**Published:** 2026-08-28

**Authors:** Ratnadeep Pramanik

**Affiliations:** Institut für Mathematik und Computergestützte Simulation, Universität der Bundeswehr München, Werner-Heisenberg-Weg 39, 85577 Neubiberg, Germany

## Abstract

**Abstract:**

Microrobotic swarms are promising candidates for targeted drug delivery in complex physiological environments, including blood, mucus, and extracellular matrices. In such biomedical scenarios, collective motion is strongly influenced by low-Reynolds-number drag, crowding, confinement, viscoelasticity, and non-Newtonian rheology. This article envisages a mechanics-based computational framework for microrobotic swarms in viscoelastic (gel-like) media. Starting from Langevin dynamics, we derive the overdamped description appropriate for microscale agents and extend it to active Brownian particles with self-propulsion, local alignment, and short-range interparticle interactions. Cohesive and purely repulsive collective regimes are represented through Lennard-Jones and Weeks-Chandler-Andersen potentials, respectively. To model gel-like rheology, we introduce a fractional Kelvin–Voigt description of drag that incorporates power-law memory effects. The resulting stochastic dynamics are mapped to an explicit Euler–Maruyama algorithm with truncated-history fractional convolution, documented stabilizers, and diagnostic observables for polarization, clustering, trajectories, and transport. Beyond the model itself, the present study synthesizes recent work on bacterial living fluids, externally driven colloidal and microrobotic swarms, fish-school hydrodynamics, neural-network control of collective patterns, and cross-scale magnetic catheter-swarm thrombus removal. That microrobotic-swarm transport in biomedical media should not be treated only as a soft-matter problem is the primary takeaway. We believe that it is rather a coupled mechanics problem in which propulsion, interaction, memory, disorder, hydrodynamic communication, and clinical deliverability must be taken into consideration. At the same time, the preliminary simulations suggest a practically important biomedical trend: whereas Newtonian transport is more prone to collective aggregation, viscoelastic transport can preserve a more distributed swarm morphology, which is encouraging for controllable delivery, broader spatial coverage, and aggregation-resistant payload transport in complex bodily fluids.

**Graphical abstract:**

**Figure:**
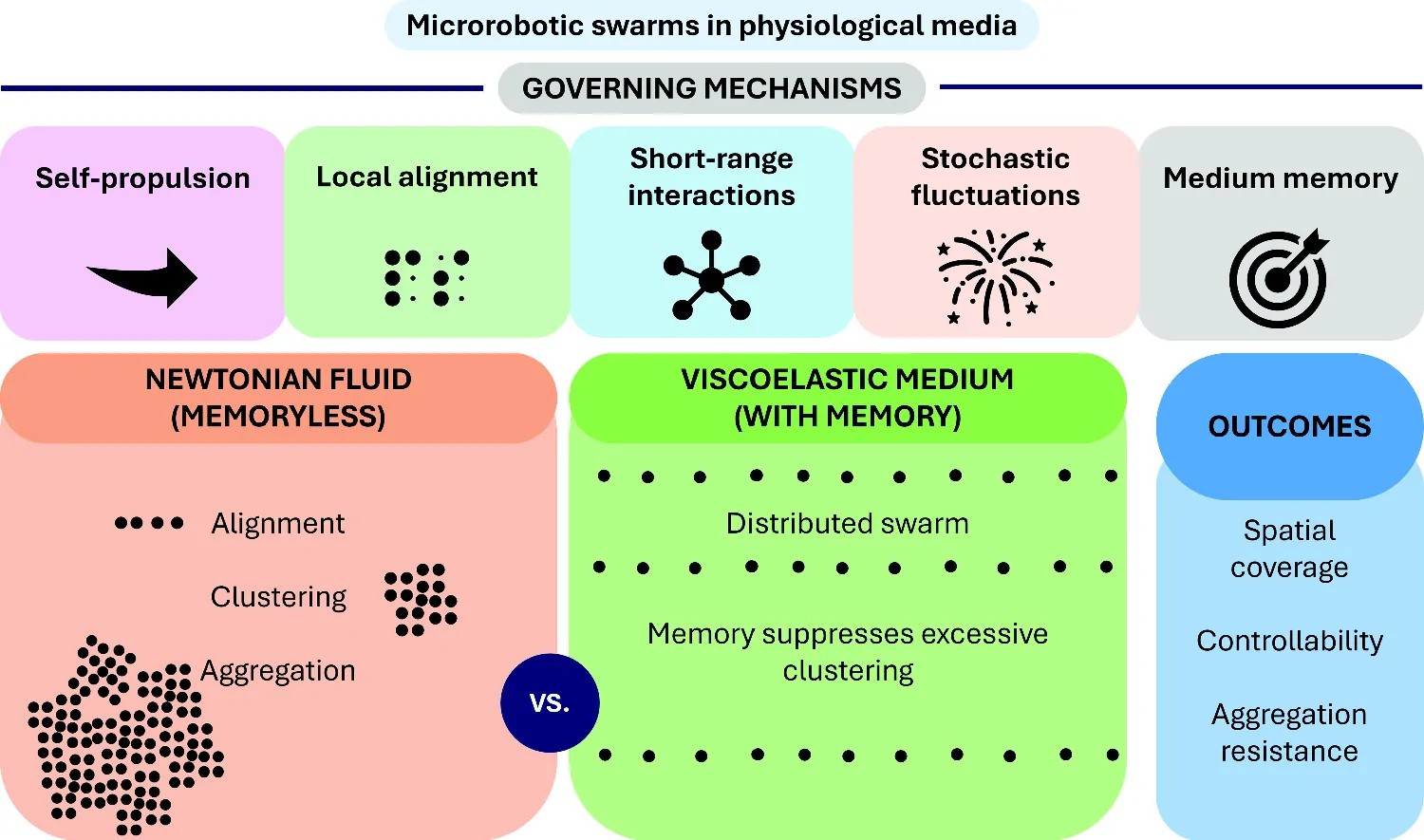

**Supplementary Information:**

The online version contains supplementary material available at https://doi.org/10.1140/epje/s10189-026-00617-4.

## Introduction

Today, medical microrobots and microrobotic swarms are being increasingly developed for targeted drug delivery, localized therapy, sensing, imaging, and minimally invasive intervention in regions that are difficult to access by conventional systemic administration or rigid instruments (catheters) [1]. Their promise lies in the ability to transport therapeutic payloads actively, increase local concentration at diseased sites, and reduce off-target exposure. From a biomedical perspective, this is relevant to navigation through vascular and capillary networks, transport toward tumor interiors, brain-directed delivery across or around blood-brain barrier constraints, and access to deep-seated lesions or confined wound environments where passive diffusion alone can be inadequate [2–6]. For example, in a recent study, Xu and co-workers proposed a cross-scale magnetic catheter-magnetic swarm strategy for precise thrombus clearance [7]. This study brought microrobotic swarms into a clinically meaningful mechanical task: localized thrombus disruption, fragment entrainment, and retrieval through an integrated catheter-swarm system.

Microrobotic swarms in complex biological media sit at the crossroad of several communities [8]: statistical physicists study nonequilibrium particles [9, 10], soft-matter researchers study gels and active fluids [11, 12], roboticists study control and actuation [13, 14], biomechanicians study transport in tissues and vessels [15, 16], and clinicians care about whether a therapeutic payload can be delivered safely and locally [17–19]. Each community uses its own words for related mechanisms that feel like jargon to others. For example, a soft-matter reader may speak of persistence, noise, mobility, and phase separation; or, a robotics reader may speak of local rules, fault tolerance, and decentralized control; or, a biomedical reader may speak of navigation, retention, penetration, safety, and retrieval. This fragmentation isolates the fields and obscures their commonalities, limiting collaborative synergy and cross-disciplinary potential. This approach restricts the ability of separate communities to leverage shared insights and mechanisms.

In this regard, the purpose of the present study is to put these languages into one mechanics-based framework. The roadmap presented here does not attempt to resolve every hydrodynamic interaction, every tissue-scale biochemical interaction, or every control-loop detail. Instead, it isolates five mechanisms that repeatedly appear across active matter and microrobotic-swarm literature [20–22]: self-propulsion, local alignment, short-range repulsion or cohesion, stochastic agitation, and medium memory. These ingredients are then tied to diagnostics that can be measured directly in simulations [23]: polarization, largest-cluster fraction, alive count, particle trajectories, and qualitative morphology. Many-particle systems repeatedly demonstrate a simple principle: if individual agents obey local rules, e.g., short-range interaction, finite sensing radius, noisy actuation, steric avoidance, delayed response, or partial alignment, collective patterns can emerge that are not explicitly programmed into any single constituent. This observation sits at the heart of swarm modeling and biomedical microrobotics [24, 25].

In equilibrium systems, emergence is often organized around free-energy minimization, entropy, and phase transitions. In active systems, by contrast, each constituent can consume or convert energy locally, so the collective state is maintained away from thermodynamic equilibrium. Once energy is injected at the particle scale, stationary states need not be Boltzmann distributed, and detailed balance is generally broken [26, 27]. At the microscale, the surrounding medium often enforces an additional simplification: the Reynolds number is small, so inertia becomes negligible compared with viscous dissipation. Purcell’s discussion of *life at low Reynolds number* made this intuition memorable, while later reviews clarified the fluid-mechanical structure of the same limit in biological and artificial microswimmers [28, 29]. Even when one is not modeling a literal swimmer with resolved hydrodynamics, the same low-Reynolds-number viewpoint motivates an overdamped equation of motion for particles embedded in a viscous (or gel-like) (micro)environment.

The stochastic foundation of such models goes back to Brownian motion. Einstein related diffusion to microscopic agitation [30]; Smoluchowski and Langevin then gave complementary kinetic and stochastic descriptions [31, 32]; and later developments in stochastic processes and Fokker-Planck theory turned these ideas into a systematic framework for nonequilibrium modeling [33, 34]. Once the stochastic baseline is in place, two ingredients dominate much of active-matter pattern formation. The first is self-propulsion, which converts a passive Brownian particle into an active one. A minimal and widely used continuum description is the ABP, where each particle moves at an approximately constant speed along a noisy heading direction [27]. The second ingredient is interaction. Some interactions are purely steric or purely repulsive, while others are cohesive and promote aggregation. The LJ potential is a classical compact model of short-range repulsion plus longer-range attraction [35]; the WCA truncation retains only the repulsive branch and is, therefore, often used when one wants excluded-volume effects without explicit attraction [36]. Even these simple choices matter enormously: attraction-driven clustering, steric jamming, flocking, traveling bands, and motility-induced phase separation can look superficially similar but arise from different mechanisms [37–39].

A second major route to collective order is alignment. Vicsek’s model showed that local heading alignment plus noise can generate long-range collective motion and nonequilibrium phase transitions [40]. In continuous-time stochastic models, the same tendency can be written as a smooth turning dynamics for particle orientation. This representation is especially convenient when one wants a model that is differentiable enough for stable numerical integration, easy to interpret physically, and straightforward to implement with vectorized array operations. The environment itself can also be an active participant in the dynamics. A Newtonian fluid responds through instantaneous viscous drag, but many gels, polymeric solutions, mucus-like media, and crowded biological environments possess broad relaxation spectra and memory. In such media, the present resistive force depends not only on the present velocity but also on the past trajectory.

Fractional constitutive models offer a compact way to represent such power-law memory and have become standard tools in anomalous diffusion and viscoelasticity [41–45]. Among the simplest and most interpretable choices is a fractional Kelvin–Voigt form, where a classical spring-dashpot picture is generalized by replacing an integer-order derivative with a fractional derivative of order $\alpha \in (0,1)$ [46]. The resulting model is still compact, yet it is rich enough to encode history-dependent drag. Physiological transport occurs in blood, mucus, interstitial spaces, extracellular matrices, and confined luminal geometries whose mechanics can be shear-thinning, viscoelastic, thixotropic, heterogeneous, or several of these simultaneously. Recent microrobot studies have shown that propulsion strategies that work in water can change substantially in complex biofluids, while biomechanics studies emphasize that tissues and extracellular matrices possess time-dependent viscoelastic behavior that directly influences transport and mechanotransduction [47–50]. Therefore, introducing memory into the drag law is not only a mathematical generalization, but it is also a biomechanics-motivated step toward more realistic transport models for *in vivo* microrobotic systems.

A central biomedical motivation of the present work is that a microrobotic drug carrier population should ideally avoid excessive aggregation, jamming, or irreversible clustering during transport, because such collective states can reduce accessible surface area, hinder penetration into confined physiological spaces, compromise controllability, and ultimately lower effective delivery efficiency at the target site. From this perspective, the comparison between Newtonian drag and viscoelastic Kelvin–Voigt-type drag is not merely constitutive; it becomes functionally relevant for drug delivery. Our simulations are, therefore, designed to test whether the surrounding medium promotes compact collective aggregation or instead supports a more spatially distributed, discrete, and transport-favorable swarm state. In this interpretation, the observation that viscoelastic memory can suppress excessive clustering and maintain more separated swarm configurations is encouraging, because it suggests that the non-Newtonian and viscoelastic character of bodily fluids may, under suitable conditions, assist rather than obstruct microrobotic delivery. This is especially relevant because real therapeutic transport and payload release occur over finite and formulation-dependent time windows, so transient collective organization, rather than only asymptotic steady state, may determine practical delivery success. In this sense, the present framework provides not only a computational mechanics perspective on emergent swarm behavior, but also an optimistic design signal for future biomedical microrobotic transport in complex physiological media.

Therefore, the scope of this perspective is not to attempt to resolve full hydrodynamic interactions between all particles, nor do we construct a fully thermodynamically consistent viscoelastic bath with noise. Instead, we present a computational roadmap whose assumptions are explicit, whose numerical stabilizers are reported rather than hidden, and whose diagnostics are simple enough to support large parameter sweeps. The framework is intended both as a tutorial entry point for young researchers and as a reproducible baseline from which more advanced models can later be built.

## Precedents and paradigms

### From local interaction rules to collective behavior

Matarić’s early work on designing emergent behaviors from local interactions is a useful conceptual starting point for the entire study [51]. The central point is that group-level coordination does not require every individual to know the full state of the group. Instead, robust collective behavior can arise when individuals respond to local cues through simple rules. That logic is directly reflected in the present model: alignment is local, interaction is pairwise, and global order is measured only after it emerges. In this sense, the computational framework is not a centralized-control model; it is a mechanical version of local-rule emergence. The Vicsek model later became the canonical minimal example of local alignment producing collective motion [40]. It shows that even point-like self-driven particles with noisy headings can undergo an order–disorder transition.

In the present study, the continuous-time alignment law is deliberately close to this tradition, but it is embedded in an overdamped force-balance setting. This allows one to place Vicsek-type consensus next to physical drag, pair potentials, diffusion, and viscoelastic memory rather than treating flocking as a purely algorithmic update rule. Another study on modeling collective behavior and pattern formation in bacterial colonies is important here because it links active-matter theory to microbial growth and colony morphology [52]. One part generalizes the Vicsek model by allowing particle speed to depend on local density, thereby connecting motility regulation with pattern formation. Another part treats growing bacterial biofilms as mechanically interacting cells that push each other as they grow. Therefore, this study reminds us that biological swarms are rarely only moving particles; they may also grow, alter their medium, consume nutrients, and change their mechanical interactions over time.

### Living fluids and bacterial active matter

Koch and Subramanian’s review on the collective hydrodynamics of swimming microorganisms provides the fluid-mechanical background for *living fluids* [53]. Their central message is that concentrated bacterial suspensions can develop coordinated motions on scales much larger than a single cell, despite negligible inertia and gravity. The key mechanism here is the coupling between swimming-induced stresses, fluid velocity disturbances, and swimmer orientation. The present paper does not resolve this hydrodynamic coupling explicitly, but it uses the same low-Reynolds-number logic. At the microscale, collective motion is shaped by instantaneous force balance and by the way the medium transmits disturbance and memory. Sokolov and co-workers demonstrated that swimming bacteria can power microscopic gears, turning disordered bacterial activity into mechanical work [54]. This study is conceptually valuable because it shows that active fluctuations are not merely noise. When constrained by geometry, they can drive useful motion.

For biomedical microrobotics, the parallel is obvious: collective activity must eventually be converted into directed transport, local mixing, retention, or mechanical action. In our computational framework, this motivates the distinction between random agitation, self-propulsion, and coherent collective order. Aranson’s review of out-of-equilibrium colloidal suspensions connects biologically active matter with engineered active colloids [55]. It discusses how externally driven colloids, self-propelled particles, and active suspensions can exhibit clustering and collective motion. The review is especially relevant here because it treats active colloids as experimental realizations of nonequilibrium statistical physics. The present paper follows the same essence by treating microrobotic agents as active soft-matter objects whose collective states can be organized by a small number of physical mechanisms.

### Field-driven colloids and magnetic microrobotic swarms

Martin and Snezhko reviewed how external fields drive self-assembly and emergent dynamics in colloidal systems [56]. Magnetic and electric fields do more than move isolated particles—they modify interactions, assemble structures, and generate time-dependent collective states. In the present framework, this literature motivates the idea that *interaction strength* and *propulsion* need not be intrinsic particle properties only. In microrobotic systems, they may be controlled externally through actuation, field amplitude, frequency, and geometry. Dobnikar, Snezhko, and Yethiraj emphasized emergent colloidal dynamics in electromagnetic fields [57]. The study focused on field-mediated collective behavior, where the medium and the external forcing together determined the accessible states. Such systems cannot be understood by simply adding a constant drive to isolated Brownian particles. They require thinking in terms of coupled interactions, boundary conditions, and collective response. This motivates our later insistence on documenting boundary conditions, cutoffs, and numerical stabilizers as part of the model specification.

Nguyen and co-workers showed that active rotation of hard shapes can generate emergent collective phenomena even without conventional attractive potentials [58]. This is a crucial lesson for interpreting simulations. If clustering appears, it does not automatically mean that explicit cohesion is present. This is because it can arise from activity, collisions, shape anisotropy, or confinement. Hence, our current framework includes both LJ and WCA cases. Comparing cohesive LJ and purely repulsive WCA simulations helps separate attraction-driven aggregation from activity-driven or steric collective states. Yigit, Alapan, and Sitti’s work on programmable collective behavior in dynamically self-assembled mobile microrobotic swarms provides a direct microrobotics link to the present study [6]. They demonstrated that microrobot collectives can be reconfigured dynamically and used as functional mobile assemblies rather than as independent particles. This motivates treating swarm morphology as a state variable in its own right.

In the present framework, clustering, polarization, and snapshot mosaics are, therefore, not merely outputs; rather, they are the quantities through which one reads whether a collective microrobotic function may be emerging. Later, they discussed cohesive self-organization of mobile microrobotic swarms [59]. Our study is particularly close to the modeling choices made here because it highlights the balance between cohesion, mobility, and collective structure. In our equations, that balance appears through the competition between self-propulsion, noise, local alignment, and LJ attraction. The cohesive swarm literature also shows why overly strong attraction is not automatically beneficial: a swarm must be coherent enough to remain functional but not so locked that it loses adaptability.

Pramanik, Verstappen, and Onck investigated the hydrodynamic interactions between magnetic soft robotic swimmers using systematically varied initial configurations [60–62]. Their study examined the emergence of three-dimensional spatiotemporal patterns and collective motion under magnetic actuation. They observed that swimmers with initial variations in the swimming direction attract each other, whereas variations in lateral positions cause repulsion. Consequently, the swimmers eventually converge into a single lateral plane and drift radially outward. In future microrobotic extensions, researchers might exploit these self-assembling configurations to coordinate collective transport or targeted delivery missions.

Wang and Zhang’s review on external power-driven microrobotic swarms is one of the clearest bridges from fundamental swarm physics to imaging-guided biomedical delivery [63]. It emphasizes swarm formation, navigation, pattern transformation, localization, and delivery as a continuous pipeline. This provides the application-level motivation for keeping the computational model interpretable. If a model is to help with imaging-guided delivery, each parameter must eventually be linked to an experimentally adjustable quantity such as field strength, actuation frequency, local viscosity, propulsion speed, or confinement. Gardi and co-workers demonstrated microrobot collectives with reconfigurable morphologies, behaviors, and functions [64]. It was concluded that morphology is not just a by-product of collective behavior; it can be programmed as a functional mode. Rings, chains, carpets, and rotating assemblies can perform different tasks.

In the present framework, this justifies using multiple diagnostics rather than a single order parameter. A high-polarization flock, a compact cluster, and a rotating mill may all be *ordered*, but they imply different biomedical capabilities. Chen and Bechinger studied the collective response of microrobotic swarms to external stimuli using programmable active colloids [65]. Their results connected collective organization to robustness: group escape can remain efficient even when only part of the population responds correctly. This is a key idea for medical microrobots, where agents may fail, lose actuation, or experience heterogeneous local environments. It motivates future versions of the present model in which particle response is heterogeneous (instead of all particles sharing the same parameters). Huaroto and co-workers studied non-uniform magnetic fields for the collective behavior of self-assembled magnetic pillars [66]. This work expands the design space from uniform actuation to spatially varying fields, which can shape collective response in more refined ways.

For the present framework, this points toward a natural extension: replacing constant parameters such as $v_0$, $K_{\textrm{align}}$, or ε by fields that vary in space and time. That would move the model closer to practical electromagnetic manipulation systems, where the actuation landscape is never perfectly uniform. Yang and co-workers’ survey of swarm microrobotics organizes the field around actuation, control, localization, and application requirements [67]. It is useful here because it clarifies why many biomedical tasks demand swarms rather than single microrobots. This is due to higher payload, imaging contrast, redundancy, and collective manipulation that scale with the number of agents. This supports the many-particle focus of the present study, where the model is not a single-particle transport model but a collective one.

### Fish schools and bioinspired underwater swarms

Ko, Lauder, and Nagpal proposed *fluid stigmergy* as a way of understanding collective motion in fish schools and bioinspired underwater robots [68]. Here, the fluid was treated not just as a source of drag but also as a medium that contains information regarding the kinematic history. Fish and robots coordinate through wakes, vortices, pressure fields, and flow sensing. The present paper does not resolve wake-mediated communication explicitly, but this literature motivates future hydrodynamic extensions. Ligman, Lund, and Fürth have provided a comprehensive review of hydrodynamic studies on fish schooling [69]. They compared physical experiments and numerical simulations and highlighted two recurring mechanisms behind energetic benefits: vortex capture and channeling. It was inferred that collective transport through fluids cannot be reduced to alignment rules alone. Spatial arrangement, phase, body flexibility, wake structure, and hydrodynamic interaction all matter. Therefore, for the present active-particle model, this means LJ/WCA-alignment simulations should be viewed as a baseline, not an exhaustive hydrodynamic description.

Zhao and Shi numerically studied two self-propelled fish-like swimmers using a muscle-contraction pre-strain model coupled to fluid–structure interaction [70]. Their study combined passive hydrodynamic interaction with active control and muscle-like actuation. They observed that an optimal collective behavior may require unequal effort among individuals. In future microrobotic extensions, one might, therefore, allow adaptive propulsion speeds or duty cycles rather than imposing one common $v_0$. Zhang and co-workers performed a three-dimensional computational study of hydrodynamic interactions in fish schools, focusing on in-line and side-by-side configurations [71]. Heydari, Hang, and Kanso mapped spatial patterns to energetic benefits in groups of flow-coupled swimmers [72]. Their study separated reciprocal and non-reciprocal flow-coupling effects in different formations. Side-by-side arrangements can distribute energetic costs more evenly, whereas in-line arrangements can favor trailing swimmers but may lose cohesion beyond a finite group size. For biomedical swarms, this suggests a general design principle: collective geometry controls not only where the agents are, but also how mechanical cost and benefit are distributed across the group.

### Inverse design of collective patterns

Kim, Lee, and Kim used deep neural networks to command emergent behaviors by learning interaction rules that generate desired global patterns [73]. Their study is relevant to the future of the present framework because it reverses the usual question. Instead of asking what pattern follows from a given interaction law, it asks what interaction law is needed to achieve a desired pattern, such as a clump, flock, or hybrid formation. This suggests a natural next step: use the mechanics-based microrobotic-swarm simulator developed here as a differentiable or surrogate-assisted engine for inverse design of biomedical swarm morphologies.

Taken together, these studies suggest several requirements for a useful baseline model. First, it must represent local rules because collective behavior emerges from local interaction rather than global prescription. Second, it must include activity and noise because active agents are not passive Brownian particles. Third, it must distinguish repulsion from cohesion because different mechanisms can produce visually similar clusters. Fourth, it must leave room for medium effects: hydrodynamic coupling, viscoelasticity, confinement, and disorder can all redirect transport. Fifth, it must be compatible with control and inverse design, since modern swarm systems are increasingly programmable. Sixth, it must retain biomedical interpretability: parameters should eventually connect to actuation, imaging, safety, delivery, and retrieval.

## Computational framework

### Simulation space

We consider *N* particles in spatial dimension $d\in \{2,3\}$ moving in a domain $\Omega =[0,L]^d$, where the position of particle *i* at time *t* is denoted by $\textbf{r}_i(t)\in \mathbb {R}^d$ (see Fig. 2a for a schematic illustration). In an overdamped model, position is the primary kinematic variable. Velocity still has physical meaning, but it is often an auxiliary quantity reconstructed from forces rather than evolved as an independent state variable. For orientation, the notation depends on the dimension. In two dimensions, it is natural to use a heading angle $\theta _i(t)\in [-\pi ,\pi ]$ and define the unit-heading vector

1 $$\begin{aligned} \textbf{n}_i(t)=\big (\cos \theta _i(t),\,\sin \theta _i(t)\big ). \end{aligned}$$

In three dimensions, one instead works directly with a unit vector $\textbf{n}_i(t)\in \mathbb {R}^3$ satisfying $\Vert \textbf{n}_i(t)\Vert =1$. Throughout most of this paper, we focus on 2D because it is computationally less expensive and visually easier to interpret, while the physical logic works equally for 3D. To keep the notation physically transparent, we explicitly name the main dimensional quantities used in the model. Here, *L* is the linear size of the computational domain, σ is the effective particle diameter or interaction length scale, $v_0$ is the self-propulsion speed, ζ is the translational drag coefficient, $\mu =1/\zeta$ is the particle mobility, $D_\text {t}$ is the translational diffusion coefficient, $D_\text {r}$ is the rotational diffusion coefficient, ε is the pair-interaction energy scale, and $\zeta _\alpha$ is the fractional memory drag coefficient. Their dimensions are as follows: *L* and σ have units of length; $v_0$ has units of length per time; ζ has units of force times time divided by length; μ has units of velocity per force; $D_\text {t}$ has units of length squared per time; $D_\text {r}$ has units of inverse time; and ε has units of energy.

### Model parameters

The parameter ζ denotes the translational drag coefficient. In a Newtonian Stokes-like setting, the drag force is proportional to velocity and opposes motion. The mobility is $\mu =1/\zeta$, so a higher drag corresponds to lower mobility. The quantity $k_BT$ is used as a noise scale. In a passive thermal system, it is the thermal energy that is related to diffusion through the Einstein relation. In an active-matter simulation, however, $k_BT$ is often better interpreted as an effective noise intensity summarizing unresolved fluctuations, environmental heterogeneity, or coarse-graining error. Self-propulsion is modeled by a constant speed $v_0$ in the heading direction $\textbf{n}_i(t)$. Alignment is controlled by a metric interaction radius $R_{\textrm{align}}$ and a rate parameter $K_{\textrm{align}}$. The larger $R_{\textrm{align}}$ is, the more neighbors contribute to the local mean direction. The larger $K_{\textrm{align}}$ is, the faster a particle turns toward that local direction. Rotational diffusion with coefficient $D_\text {r}$ randomizes headings and, therefore, competes directly with alignment.

We use LJ or WCA interactions with energy scale ε and length scale σ. In a soft-potential model such as LJ, σ can be interpreted as an effective particle diameter: it marks the scale at which short-range repulsion becomes significant and the potential changes character. The parameter ε sets the overall interaction strength. A cutoff distance $r_\text {c}$ truncates pair interactions for efficiency. For WCA, the canonical choice is $r_c=2^{1/6}\sigma$, which cuts the potential at its minimum so only the repulsive branch remains. For truncated LJ, a common practical choice is $r_\text {c}=2.5\sigma$.

To model gel memory, we introduce a fractional order $\alpha \in (0,1)$ and a viscoelastic strength parameter $\zeta _\alpha$. The order α controls how slowly the memory kernel decays. A small value of α corresponds to a long memory tail. As α approaches one, the memory becomes more short-ranged and more dashpot-like. The coefficient $\zeta _\alpha$ controls the magnitude of this history-dependent drag. Note that we do not emphasize a single fixed numerical parameter set here. Instead, the framework is intended to illustrate how density, activity, alignment, interaction strength, and viscoelastic memory may be varied systematically within a transparent computational setting. The parameter choices used to generate the illustrative figures should, therefore, be interpreted as representative rather than universal, and the emphasis is placed on qualitative trends and physically interpretable transitions in collective behavior.

### From Newton’s law to overdamped Langevin dynamics

Consider a particle of mass *m*, position $\textbf{r}_i(t)$, and velocity $\textbf{v}_i(t)=\dot{\textbf{r}}_i(t)$. Newton’s second law states

2 $$\begin{aligned} m\dot{\textbf{v}}_i(t)=\textbf{F}_i(\textbf{r}(t),t), \end{aligned}$$

where $\textbf{F}_i$ may include external forcing, pair interactions, confinement, and any other mechanical components. At low Reynolds numbers, viscous damping dominates inertia. The simplest phenomenological description of this effect is linear drag,

3 $$\begin{aligned} \textbf{F}_i^{\textrm{drag}}(t)=-\zeta \,\textbf{v}_i(t), \end{aligned}$$

with ζ>0. The minus sign ensures that drag opposes motion. This is the standard Newtonian-fluid approximation and is the correct first model before introducing memory [28, 29]. Microscopic particles are continuously bombarded by unresolved degrees of freedom of the environment. Langevin’s idea is used to model this unresolved forcing by a random term $\boldsymbol{\eta }_i(t)$:

4 $$\begin{aligned} m\dot{\textbf{v}}_i(t)=\textbf{F}_i(\textbf{r}(t),t)-\zeta \textbf{v}_i(t)+\boldsymbol{\eta }_i(t). \end{aligned}$$

The standard white-noise idealization assumes

5 $$\begin{aligned}  &   \langle \eta _{i\alpha }(t)\rangle =0,\qquad \langle \eta _{i\alpha }(t)\eta _{j\beta }(t')\rangle =2\zeta k_BT\,\nonumber \\  &   \quad \delta _{ij}\delta _{\alpha \beta }\delta (t-t'). \end{aligned}$$

In a passive equilibrium setting, the pre-factor is required by the fluctuation-dissipation balance [74]. In active coarse-grained models, the same algebraic form is often retained even when $k_BT$ is interpreted as an effective noise scale. It is more precise to write the model using Wiener processes:

6 $$\begin{aligned} d\textbf{r}_i&= \textbf{v}_i\,dt, \end{aligned}$$

7 $$\begin{aligned} m\,d\textbf{v}_i&= \textbf{F}_i(\textbf{r},t)\,dt-\zeta \textbf{v}_i\,dt+\sqrt{2\zeta k_BT}\,d\textbf{W}_i(t). \end{aligned}$$

The inertial relaxation time is $\tau _m=m/\zeta$. If the timescale of interest is much larger than $\tau _m$, velocity adjusts rapidly compared with the slow motion we care about. Taking the overdamped or Smoluchowski limit gives

8 $$\begin{aligned}  &   d\textbf{r}_i=\mu \textbf{F}_i(\textbf{r},t)\,dt+\sqrt{2D_t}\,d\textbf{W}_i(t),\qquad \mu =\frac{1}{\zeta },\nonumber \\  &   \quad D_t=\frac{k_BT}{\zeta }. \end{aligned}$$

This positional stochastic differential equation is the fundamental passive baseline used henceforth. The corresponding Fokker-Planck equation for the additive-noise case is

9 $$\begin{aligned} \partial _t P=-\sum _{i=1}^N\nabla _{\textbf{r}_i}\cdot (\mu \textbf{F}_iP)+D_t\sum _{i=1}^N\Delta _{\textbf{r}_i}P. \end{aligned}$$

Although we do not solve this partial differential equation directly, it gives valuable intuition: noise broadens distributions, while drift concentrates (or transports) them. The standard Euler–Maruyama discretization is

10 $$\begin{aligned}  &   \textbf{r}_i^{n+1}=\textbf{r}_i^n+\mu \textbf{F}_i(\textbf{r}^n,t_n)\Delta t+\sqrt{2D_t\Delta t}\,\boldsymbol{\xi }_i^n, \nonumber \\  &   \quad \Delta \textbf{W}_i^n = \textbf{W}_i(t_{n+1})-\textbf{W}_i(t_n) = \sqrt{\Delta t}\,\boldsymbol{\xi }_i^n, \end{aligned}$$

so that $d\textbf{W}_i(t)$ in the continuous stochastic differential equation is approximated over one time step by $\Delta \textbf{W}_i^n$. Thus, the deterministic drift terms scale as Δt, whereas the stochastic Brownian increments scale as $\sqrt{\Delta t}$.

### Interparticle potentials and forces

For two particles *i* and *j*, define $\textbf{r}_{ij}=\textbf{r}_i-\textbf{r}_j,\qquad r_{ij}=\Vert \textbf{r}_{ij}\Vert$. If the interaction is pairwise additive, the total potential energy is

11 $$\begin{aligned} U(\textbf{r})=\sum _{1\le i<j\le N}U(r_{ij}), \end{aligned}$$

and the force on particle *i* is

12 $$\begin{aligned} \textbf{F}_i(\textbf{r})=-\nabla _{\textbf{r}_i}U(\textbf{r})=\sum _{j\ne i}\textbf{F}_{ij}. \end{aligned}$$

The LJ potential is

13 $$\begin{aligned} U_{\textrm{LJ}}(r)=4\varepsilon \left[ \left( \frac{\sigma }{r}\right) ^{12}-\left( \frac{\sigma }{r}\right) ^6\right] . \end{aligned}$$

The $r^{-12}$ term gives strong short-range repulsion; the $r^{-6}$ term gives intermediate-range attraction. Upon differentiation, we obtain

14 $$\begin{aligned} \textbf{F}_{ij}=24\varepsilon \left[ 2\left( \frac{\sigma }{r_{ij}}\right) ^{12}-\left( \frac{\sigma }{r_{ij}}\right) ^6\right] \frac{\textbf{r}_{ij}}{r_{ij}^2}. \end{aligned}$$

The WCA potential is the purely repulsive branch of LJ:

15 $$\begin{aligned} U_{\textrm{WCA}}(r)= {\left\{ \begin{array}{ll} 4\varepsilon \left[ \left( \dfrac{\sigma }{r}\right) ^{12}-\left( \dfrac{\sigma }{r}\right) ^6\right] +\varepsilon , &  r<2^{1/6}\sigma ,\\ 0, &  r\ge 2^{1/6}\sigma . \end{array}\right. }\nonumber \\ \end{aligned}$$

**Fig. 1:**
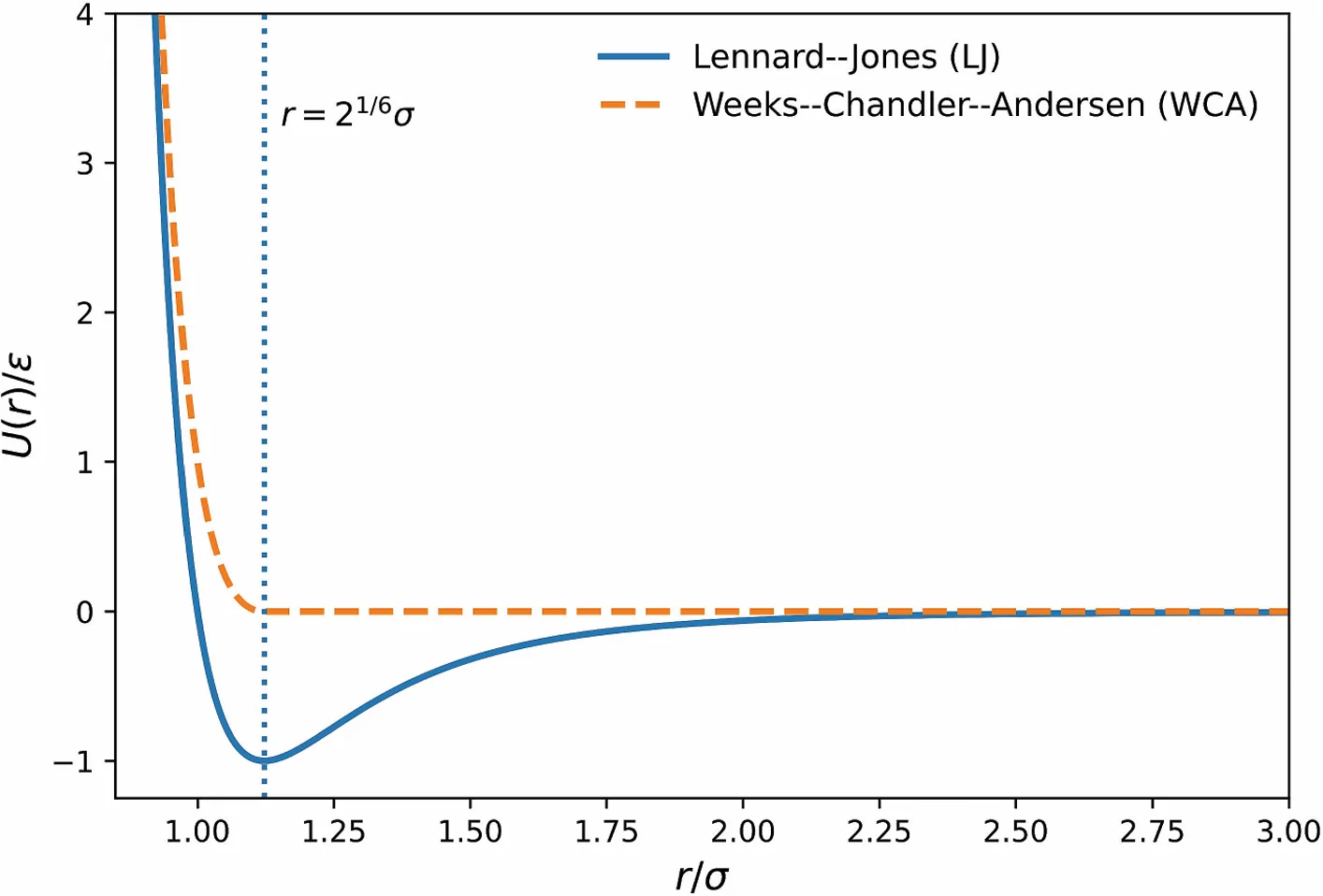
Dimensionless LJ and WCA potentials. The LJ potential combines short-range repulsion with longer-range attraction, while the WCA potential is shifted and truncated at the LJ minimum, $r=2^{1/6}\sigma$, retaining only the purely repulsive branch

Figure 1 illustrates the difference between the two interaction laws. The LJ potential contains both a steep short-range repulsive core and a longer-range attractive tail. By contrast, the WCA potential is obtained by shifting and truncating the LJ potential at its minimum, $r=2^{1/6}\sigma$, so that only the repulsive branch remains. This distinction is important for interpreting collective states: clustering in the LJ case can be promoted directly by cohesion, whereas clustering in the WCA case cannot be attributed to pairwise attraction.

Because the added +ε is constant, it does not change the force on the interacting branch. Comparing LJ and WCA in the same framework is useful because it separates explicit attraction from activity-driven clustering.

### Active motion and local alignment

The ABP position equation is

16 $$\begin{aligned} d\textbf{r}_i = \left( v_0\textbf{n}_i(t) + \mu \textbf{F}_i(\textbf{r}(t)) \right) dt + \sqrt{2D_t}\,d\textbf{W}_i(t). \end{aligned}$$

The self-propulsion drift is not the gradient of a potential and therefore drives the system out of equilibrium [25, 27]. In two dimensions, a purely diffusive heading satisfies

17 $$\begin{aligned} d\theta _i = \sqrt{2D_r}\,dW_i^{(r)}(t). \end{aligned}$$

This equation defines the bare orientational persistence time

18 $$\begin{aligned} \tau _r=D_r^{-1}. \end{aligned}$$

For the standard Newtonian, non-interacting ABP, this corresponds to a persistence length of order

19 $$\begin{aligned} \ell _p\sim \frac{v_0}{D_r}. \end{aligned}$$

To include Vicsek-type consensus, we define the neighbor set

20 $$\begin{aligned} \mathcal {N}_i(t) = \left\{ j\ne i: \left\| \textbf{r}_j(t)-\textbf{r}_i(t) \right\| < R_{\textrm{align}} \right\} . \end{aligned}$$

The mean local heading is computed by averaging unit-heading vectors rather than the angles themselves:

21 $$\begin{aligned} \bar{\theta }_i = \textrm{atan2} \left( \sum _{j\in \mathcal {N}_i}\sin \theta _j, \sum _{j\in \mathcal {N}_i}\cos \theta _j \right) . \end{aligned}$$

If $\mathcal {N}_i$ is empty, we set $\bar{\theta }_i=\theta _i$.

The continuous-time alignment rule is

22 $$\begin{aligned} d\theta _i = K_{\textrm{align}} \sin (\bar{\theta }_i-\theta _i)\,dt + \sqrt{2D_r}\,dW_i^{(r)}(t). \end{aligned}$$

The full Newtonian active interacting model is therefore

23 $$\begin{aligned} d\textbf{r}_i&= \left( v_0\textbf{n}_i(t) + \mu \textbf{F}_i(\textbf{r}(t)) \right) dt + \sqrt{2D_t}\,d\textbf{W}_i(t), \end{aligned}$$

24 $$\begin{aligned} d\theta _i&= K_{\textrm{align}} \sin (\bar{\theta }_i-\theta _i)\,dt + \sqrt{2D_r}\,dW_i^{(r)}(t). \end{aligned}$$

The principal competitions are between self-propulsion and translational diffusion, quantified by $v_0\sigma /D_t$; alignment and rotational diffusion, quantified by $K_{\textrm{align}}/D_r$; interaction strength and stochastic agitation, quantified by $\varepsilon /k_BT$; and the alignment radius relative to the characteristic interparticle spacing.

To distinguish intrinsic orientational persistence from the persistence of the realized translational trajectory, we define the heading time-correlation function

25 $$\begin{aligned} C_n(\tau ) = \left\langle \textbf{n}_i(t+\tau )\cdot \textbf{n}_i(t) \right\rangle _{i,t}, \end{aligned}$$

where the average is taken over particles and time origins. For a two-dimensional non-interacting particle without alignment, $K_{\textrm{align}}=0$, the angular increment $\theta _i(t+\tau )-\theta _i(t)$ is Gaussian with variance $2D_r\tau$. Consequently,

26 $$\begin{aligned} C_n(\tau ) = \left\langle \cos \!\left[ \theta _i(t+\tau )-\theta _i(t) \right] \right\rangle = \exp (-D_r\tau ). \end{aligned}$$

Thus, the bare orientational correlation decays exponentially over the characteristic time $D_r^{-1}$.

The fractional drag term introduced in Sect. 3.6 does not appear directly in the orientation equation and therefore does not directly alter this bare rotational decorrelation time. In an interacting and locally aligned system, however, fractional drag can influence $C_n(\tau )$ indirectly by modifying particle positions and, consequently, the time-dependent neighbor sets $\mathcal {N}_i(t)$ entering the alignment rule. A simple closed-form expression is generally unavailable in this case, but the correlation can be calculated from uniformly sampled heading histories using

27 $$\begin{aligned}  &   \widehat{C}_n(\tau _m) = \frac{1}{N(N_\text {s}-m)} \sum _{i=1}^{N} \sum _{q=0}^{N_\text {s}-m-1} \textbf{n}_i(t_{q+m}) \cdot \nonumber \\  &   \quad \textbf{n}_i(t_q), \end{aligned}$$

where $N_\text {s}$ is the number of stored time samples, *m* is the discrete time-lag index, and

28 $$\begin{aligned} \tau _m=m\Delta t_{\textrm{samp}} \end{aligned}$$

is the corresponding time lag for a sampling interval $\Delta t_{\textrm{samp}}$. The fractional term therefore primarily modifies the persistence of the realized translational motion and sustained particle transport, while any change in orientational correlations arises indirectly through the evolving interaction and alignment neighborhoods.

### Fractional Kelvin–Voigt extension for viscoelastic gels

The Newtonian-drag model assumes that the resistive force depends only on the instantaneous velocity,

29 $$\begin{aligned} \textbf{F}^{\textrm{drag}}(t)=-\zeta \dot{\textbf{r}}(t). \end{aligned}$$

This is appropriate for a purely viscous solvent with no memory: once the particle velocity changes, the drag force changes immediately. Many biological and gel-like media, however, are not purely Newtonian. Mucus, polymer networks, extracellular matrices, and crowded soft biological materials can store elastic deformation and relax it only gradually. Their present mechanical response can, therefore, depend on how the material was deformed in the past [75].

A useful starting point is the classical Kelvin–Voigt model. In one dimension, it writes the stress as

30 $$\begin{aligned} \sigma (t)=E\varepsilon (t)+\eta \dot{\varepsilon }(t), \end{aligned}$$

where *E* is an elastic modulus, η is a viscosity, ε(t) is the strain, and $\dot{\varepsilon }(t)$ is the ordinary strain rate. This model may be viewed as a spring and a dashpot acting in parallel. The elastic term stores deformation, while the dashpot term dissipates energy instantaneously. In this classical form, the dashpot corresponds to an integer-order derivative, i.e., the case α=1.

The limitation of the classical Kelvin–Voigt model is that it contains only a single, local-in-time viscous rate. Many gels and soft biological materials instead show broad relaxation spectra and approximately power-law memory. In such cases, the material does not forget its deformation history exponentially through one relaxation time; rather, past deformation rates may contribute over a wide range of times. Fractional rheology provides a compact way to encode this behavior by replacing the ordinary first derivative with a derivative of non-integer order $\alpha \in (0,1)$ [43, 46]. The fractional Kelvin–Voigt form is then written as

31 $$\begin{aligned} \sigma (t)=E\varepsilon (t)+\eta _\alpha \,{}^C D_t^\alpha \varepsilon (t), \end{aligned}$$

where $\eta _\alpha$ is a fractional viscosity-like coefficient and ^C^$D_t^\alpha$ is the Caputo fractional derivative.

For a sufficiently smooth scalar function *f*(*t*), the Caputo derivative is defined as

32 $$\begin{aligned} {}^C D_t^\alpha f(t)= \frac{1}{\Gamma (1-\alpha )} \int _0^t \frac{f'(s)}{(t-s)^\alpha }\,ds, \qquad 0<\alpha <1.\nonumber \\ \end{aligned}$$

Here, the prime denotes the ordinary derivative with respect to the past time variable, i.e., $f^{\prime }\left(s\right)=df\left(s\right)/ds$. The restriction 0<α<1 is used because Eq. (32) is the standard Caputo representation for a derivative order between zero and one. The classical Kelvin–Voigt dashpot with α=1 is, therefore, not included inside this integral expression; instead, it is recovered as the ordinary first-derivative limiting case.

Equation (32) also shows why the fractional derivative naturally represents memory. It can be written as a convolution over the past rate of change,

33 $$\begin{aligned} {}^C D_t^\alpha f(t)= &   \int _0^t K_\alpha (t-s) f'(s)\,ds, \qquad K_\alpha (t-s)\nonumber \\= &   \frac{(t-s)^{-\alpha }}{\Gamma (1-\alpha )}. \end{aligned}$$

Thus, the fractional derivative is not simply a formal derivative of non-integer order. It is a history integral with a power-law memory kernel $K_\alpha$. Recent motion contributes strongly, but older motion is not forgotten abruptly. Instead, its influence decays algebraically. The parameter α controls this memory decay: smaller α corresponds to a slower-decaying memory kernel, whereas values closer to one approach a more local, dashpot-like response.

In the present particle model, we use this idea at the level of a coarse-grained drag law. The Newtonian solvent-like part of the resistance is retained through the ordinary drag coefficient ζ, while the gel-like memory contribution is represented by a fractional drag coefficient $\zeta _\alpha$. Since ^C^$D_t^\alpha \textbf{r}$ has dimensions of length divided by time$^\alpha$, the fractional drag coefficient $\zeta _\alpha$ has dimensions of force times time$^\alpha$ divided by length. The resulting generalized force balance is written as

34 $$\begin{aligned}  &   \zeta \dot{\textbf{r}}_i(t) + \zeta _\alpha \,{}^C D_t^\alpha \textbf{r}_i(t) = \zeta v_0\textbf{n}_i(t) + \textbf{F}_i(\textbf{r}(t))\nonumber \\  &   \quad + \zeta \sqrt{2D_t}\,\boldsymbol{\eta }_i(t). \end{aligned}$$

The right-hand side contains the active propulsion force written in overdamped form, $\zeta v_0\textbf{n}_i(t)$, the interaction force $\textbf{F}_i$, and the stochastic forcing term $\zeta \sqrt{2D_t}\,\boldsymbol{\eta }_i(t)$, where $\boldsymbol{\eta }_i(t)$ denotes Gaussian white noise. Dividing by ζ and writing $\mu =1/\zeta$ gives

35 $$\begin{aligned} \dot{\textbf{r}}_i(t)= &   v_0\textbf{n}_i(t) + \mu \textbf{F}_i(\textbf{r}(t)) - \mu \zeta _\alpha \,{}^C D_t^\alpha \textbf{r}_i(t) \nonumber \\  &   + \sqrt{2D_t}\,\boldsymbol{\eta }_i(t). \end{aligned}$$

The presence of two dissipative terms in Eq. (34) is intentional and should not be interpreted as double-counting the same frictional mechanism. The ordinary term $\zeta \dot{\textbf{r}}_i(t)$ represents the instantaneous solvent-like viscous resistance, which is local in time and responds only to the current particle velocity. The fractional term $\zeta _\alpha \,$^C^$D_t^\alpha \textbf{r}_i(t)$ represents the delayed resistance associated with the gel-like or polymeric microstructure of the surrounding medium. Mechanically, this is analogous to writing the drag as the sum of an instantaneous dashpot contribution and a history-dependent memory contribution acting in parallel. The limiting case $\zeta _\alpha =0$ recovers the usual Newtonian overdamped ABP model. Conversely, increasing $\zeta _\alpha$ strengthens the memory-dependent resistance. If α approaches one, the fractional contribution becomes more local and dashpot-like, while for smaller α the resistance retains a longer memory of past motion.

The physical meaning of the fractional term is, therefore, a history-dependent resistance to motion. If a particle has moved persistently in one direction, the medium retains a memory of that motion and resists the continuation of the same trend. This can slow cluster coarsening, delay structural relaxation, introduce path dependence, and modify the time scale on which dense aggregates form or dissolve. In the present work, the fractional term is, therefore, used not as a fully resolved constitutive model of a specific biological gel, but as a minimal computational mechanism for asking how medium memory may alter collective active-particle dynamics.

A fully equilibrium-consistent viscoelastic bath would generally require colored noise related to the memory friction through a generalized fluctuation-dissipation theorem [74]. We do not pursue that more detailed route here. Instead, we keep the stochastic forcing as an effective white-noise perturbation and isolate the role of memory in the drag law. This choice keeps the model transparent and suitable for the perspective-style aim of the manuscript, while still introducing the central mechanical feature missing from a purely Newtonian overdamped ABP model.

### Discretization and algorithmic implementation

For the Newtonian active model, Euler–Maruyama gives

36 $$\begin{aligned} \textbf{r}_i^{n+1}=\textbf{r}_i^n+\left( v_0\textbf{n}_i^n+\mu \textbf{F}_i(\textbf{r}^n)\right) \Delta t+\sqrt{2D_t\Delta t}\boldsymbol{\xi }_i^n. \nonumber \\ \end{aligned}$$

The heading update is

37 $$\begin{aligned} \theta _i^{n+1}=\theta _i^n+K_{\textrm{align}}\sin (\bar{\theta }_i^n-\theta _i^n)\Delta t+\sqrt{2D_r\Delta t}\xi _{r,i}^n,\nonumber \\ \end{aligned}$$

followed by wrapping into $[-\pi ,\pi ]$. Under periodic boundaries, pairwise distances must be computed using the minimum-image convention:

38 $$\begin{aligned} \textbf{r}_{ij}=\textbf{r}_i-\textbf{r}_j-L\,\textrm{round}\left( \frac{\textbf{r}_i-\textbf{r}_j}{L}\right) . \end{aligned}$$

To implement the fractional term, we use a truncated Grünwald-Letnikov-type history convolution on the uniform time grid $t_n=n\Delta t$. The reason for this form follows from the fractional-binomial expansion of a derivative operator. For an ordinary first derivative, the derivative at the current time depends only on a local finite difference. For a fractional derivative, however, the derivative at $t_n$ depends on a weighted sum of previous states. The weights are fractional-binomial coefficients,

39 $$\begin{aligned} w_k=(-1)^k{\alpha \atopwithdelims ()k}, \end{aligned}$$

which gives $w_0=1$, $w_1=-\alpha$, $w_2=\alpha (\alpha -1)/2$, and so on. In the implementation, these coefficients are generated recursively as

40 $$\begin{aligned}  &   w_0=1,\qquad w_k=\left( 1-\frac{\alpha +1}{k}\right) w_{k-1}, \nonumber \\  &   \quad k=1,2,\ldots ,M-1. \end{aligned}$$

This recursive form is numerically convenient because it avoids repeated evaluation of generalized binomial coefficients.

The Caputo derivative has the useful property that the derivative of a constant is zero. To retain this property in the discrete approximation, the initial position is subtracted from the history terms. Thus, at time $t_n$, we approximate

41 $$\begin{aligned}  &   {}^C D_t^\alpha \textbf{r}_i(t_n) \approx \mathcal {D}_\alpha \textbf{r}_i^n = \frac{1}{(\Delta t)^\alpha } \sum _{k=0}^{K_n} w_k\left( \textbf{r}_i^{n-k}-\textbf{r}_i^0\right) ,\nonumber \\  &   \quad K_n=\min (n,M-1). \end{aligned}$$

Here, $\mathcal {D}_\alpha \textbf{r}_i^n$ is not a new continuum operator. It denotes the discrete truncated-history approximation to the Caputo derivative ^C^$D_t^\alpha \textbf{r}_i(t_n)$. The factor $(\Delta t)^{-\alpha }$ appears because a derivative of order α has units of time$^{-\alpha }$. The integer *M* sets the maximum number of stored history terms, so the memory window is $T_{\textrm{mem}}=M\Delta t$.

With this definition, the explicit Euler–Maruyama update for the fractional viscoelastic active-particle model becomes

42 $$\begin{aligned} \textbf{r}_i^{n+1}= &   \textbf{r}_i^n + \left( v_0\textbf{n}_i^n + \mu \textbf{F}_i(\textbf{r}^n) - \mu \zeta _\alpha \mathcal {D}_\alpha \textbf{r}_i^n \right) \Delta t \nonumber \\  &   + \sqrt{2D_t\Delta t}\,\boldsymbol{\xi }_i^n. \end{aligned}$$

Therefore, Eq. (42) is simply the Euler–Maruyama discretization of Eq. (35), with the continuum Caputo derivative replaced by the discrete approximation defined in Eq. (41).

### Boundary conditions, initialization, and computational strategy

Reflecting boundary conditions are used here only as an idealized numerical confinement condition. Their role is to keep particles inside the computational domain by reflecting an attempted displacement at the boundary. They should not be interpreted as a realistic wall model for active particles because they do not resolve steric wall interactions, hydrodynamic wall coupling, adhesion, surface roughness, or experimentally observed accumulation near solid boundaries. Thus, in the present simulations, reflecting boundaries prevent particle loss from the finite box and allow us to compare Newtonian and viscoelastic transport under the same confinement rule.

For this reason, boundary-condition effects are treated cautiously. Periodic boundaries are useful when one wants to approximate a bulk-like system without walls. Reflecting boundaries are useful when one wants a closed finite domain, but they may artificially influence particle distributions near the edges. Absorbing or removal-type boundaries can be used when particles leaving the domain should be interpreted as lost, escaped, or no longer available for the collective transport process. The choice of boundary condition is therefore not only a numerical detail; it changes the physical interpretation of the simulation.

### Nondimensional control parameters

The global polarization is

43 $$\begin{aligned} \mathcal {P}(t)=\left\| \frac{1}{N_a(t)}\sum _{i\in \mathcal {A}(t)}\textbf{n}_i(t)\right\| , \end{aligned}$$

where $\mathcal {A}(t)$ is the set of alive particles and $N_a(t)=|\mathcal {A}(t)|$. Values near one indicate global heading alignment, while values near zero indicate orientational disorder. To quantify clustering, we define a graph whose vertices are alive particles and whose edges connect particles separated by less than $R_{\textrm{cluster}}$. The largest connected component fraction is

44 $$\begin{aligned} C_{\max }(t)=\frac{|\mathcal {C}_{\max }(t)|}{N_a(t)}. \end{aligned}$$

A practical default is $R_{\textrm{cluster}}=1.5\sigma$, though sensitivity to this threshold should be checked. The most useful dimensionless groups are

45 $$\begin{aligned}  &   \rho ^*=\frac{N\sigma ^2}{L^2},\qquad \textrm{Pe}=\frac{v_0\sigma }{D_t},\qquad \Lambda =\frac{K_{\textrm{align}}}{D_r},\nonumber \\  &   \quad \Xi =\frac{\varepsilon }{k_BT}. \end{aligned}$$

Across representative parameter choices, activity may be taken sufficiently strong to sustain persistent transport, and alignment may be chosen to compete with rotational noise. Furthermore, attraction may be strong enough to permit clustering in the Newtonian case, and viscoelastic memory may be chosen to visibly modify collective organization. Broad regimes then include disordered gas-like motion, compact clusters, coarsening aggregates, coherent flocks, traveling bands, WCA-based activity-driven clusters, and memory-modified transients.

### Preliminary results

**Fig. 2:**
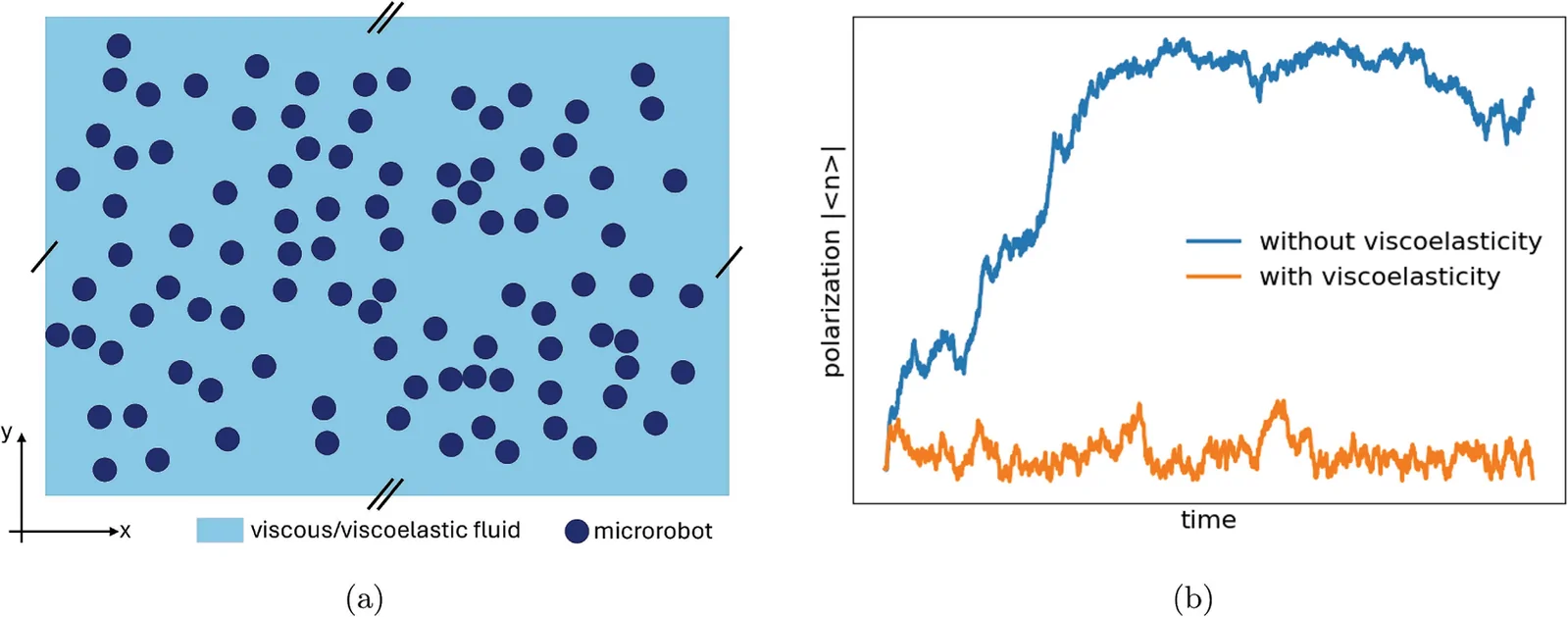
**a** Schematic representation of a microrobotic swarm in a viscous (or viscoelastic) medium. The domain of interest is periodic in the x- and y-axes: the boundaries (edges) indicate periodicity. **b** Qualitative comparison of polarization histories for Newtonian and viscoelastic transport. The Newtonian case exhibits stronger collective ordering (consistent with enhanced clustering and flock-like organization), whereas the viscoelastic Kelvin–Voigt medium maintains lower polarization and a more spatially distributed swarm state

The results presented here are intentionally preliminary and qualitative in character, consistent with the broader aim of the paper as a computational perspective rather than a parameter-sweep article. The central purpose of these illustrative simulations is not to establish a detailed phase diagram, but to highlight the strong mechanistic contrast between swarm transport in a Newtonian environment and swarm transport in a viscoelastic, non-Newtonian environment represented here through a fractional Kelvin–Voigt drag law. In the biomedical context motivating this work, this contrast is especially important because bodily fluids and soft physiological surroundings are rarely perfectly Newtonian, and their rheological memory may alter collective transport in ways that are directly relevant to drug-delivery performance.

The simulations shown in this section use the ABP framework described above, with self-propulsion, rotational diffusion, local alignment, stochastic translational noise, and short-range pair interactions. Both LJ and WCA interactions are considered in the illustrative results, as indicated explicitly in the corresponding figure captions and panel labels. This distinction is important. The LJ potential contains an attractive tail and can therefore promote explicit cohesion. By contrast, the WCA potential is purely repulsive, so clustering observed in WCA simulations cannot be attributed to pairwise attraction. In the WCA case, clustering is instead interpreted as activity-driven or MIPS-like: persistent self-propulsion and finite-density collisions cause particles to accumulate, and this accumulation can further reduce escape from dense regions, creating a positive feedback between local density and motility. The LJ potential may enhance, sharpen, or modify such aggregation, but the presence of WCA clustering demonstrates that cohesive attraction is not required for cluster formation in the active system.

The suppression of clustering in the viscoelastic simulations can be understood from the fractional Kelvin–Voigt memory term in Eq. (35). In a Newtonian medium, the drag is instantaneous. Once a particle orientation persists over a finite time, self-propulsion can carry the particle coherently into a dense region. At finite density, collisions, steric slowing, alignment, and repeated encounters increase the residence time of particles inside clusters. This is the standard physical route toward MIPS-like aggregation in ABPs: particles accumulate where their effective escape rate is reduced. In contrast, the viscoelastic fractional term introduces a history-dependent resistance that depends on the previous trajectory of each particle. Persistent directed motion is penalized by the memory of past displacement, so particles are less able to maintain a sustained inward flux into dense regions. In this sense, viscoelasticity reduces the persistence of the realized translational motion and the sustained inward particle flux, without directly changing the bare orientational decorrelation time $D_r^{-1}$. It thereby weakens the density-motility feedback that drives MIPS-like clustering and delays or suppresses compact aggregate growth.

Thus, viscoelasticity does not remove interparticle forces and does not make the particles passive. Rather, it modifies the mobility pathway through which activity, collisions, alignment, and pair interactions generate collective aggregation. For WCA interactions, this means that the memory term suppresses activity-driven, MIPS-like clustering even when there is no cohesive attraction. For LJ interactions, the same memory term competes with both activity-driven accumulation and the additional attractive contribution of the LJ tail. Across the representative simulations shown here, this leads to a more spatially distributed swarm morphology in viscoelastic media than in the corresponding Newtonian cases (Table 1).

**Table 1:** Simulation parameters used for the illustrative Newtonian and viscoelastic fractional Kelvin–Voigt swarm simulations

Quantity	Symbol	Value used
Spatial dimension	*d*	2
Number of particles	*N*	1000
Domain size	*L*	100.0
Boundary condition	–	periodic, reflecting, or absorbing, as indicated in the figure captions/panels
Interaction potential	–	LJ or WCA, as indicated in the figure captions/panels
Thermal/noise scale	$k_BT$	0.01
Translational drag coefficient	ζ	1.0
Mobility	$\mu =1/\zeta$	1.0
Translational diffusion coefficient	$D_\text {t}=k_BT/\zeta$	0.01
Self-propulsion speed	$v_0$	1.0
Alignment radius	$R_{\textrm{align}}$	2.5
Alignment strength	$K_{\textrm{align}}$	0.5
Rotational diffusion coefficient	$D_\text {r}$	0.1
Interaction energy scale	ε	1.0
Interaction length scale	σ	1.0
LJ cutoff radius	$r_\text {c}$	2.5σ
WCA cutoff radius	$r_\text {c}$	$2^{1/6}\sigma$
Fractional order, viscoelastic cases	α	0.5
Fractional drag coefficient, viscoelastic cases	$\zeta _\alpha$	0.05
Memory length, viscoelastic cases	*M*	256
Memory window	$T_{\textrm{mem}}=M\Delta t$	0.256
Time step	Δt	0.001
Number of time steps	–	1000
Final simulation time	*T*	1.0
Displayed snapshot times	–	Fig. 3: $t/T=\{0.001,\,0.01,\,0.1,\,1\}$; Figs. 4 and 5: $t/T=\{0,\,0.2,\,0.4,\,0.6,\,0.8,\,1\}$
Initialization	–	lattice with jitter; additional tests use Gaussian and uniform initializations where indicated
Jitter amplitude	–	0.05σ
Soft-core regularization	–	enabled
Minimum pair distance	$r_{\min }$	0.85σ
Force clipping	–	enabled
Maximum force magnitude	$F_{\max }$	200.0
Random seed	–	0

LJ or WCA interactions are used as indicated in the individual figure captions and panel labels. The Newtonian and viscoelastic cases use the same base active-particle parameters; the viscoelastic case additionally includes the fractional memory parameters α=0.5, $\zeta _\alpha =0.05$, and M=256

Figure 2b shows that the polarization histories differ noticeably when viscoelasticity is introduced. In the Newtonian case, the larger polarization indicates a stronger tendency toward collective ordering, coherent motion, and the emergence of clustered or flock-like organization. By contrast, in the viscoelastic case, the polarization remains lower and less suggestive of compact coherent aggregation. This is consistent with the interpretation that history-dependent drag disrupts or weakens the development of strongly collective ordered states. Importantly, the temporal curves also indicate that the system response is emergent and evolving rather than trivially stationary, which is precisely the kind of transient behavior that matters in realistic transport settings.

**Fig. 3:**
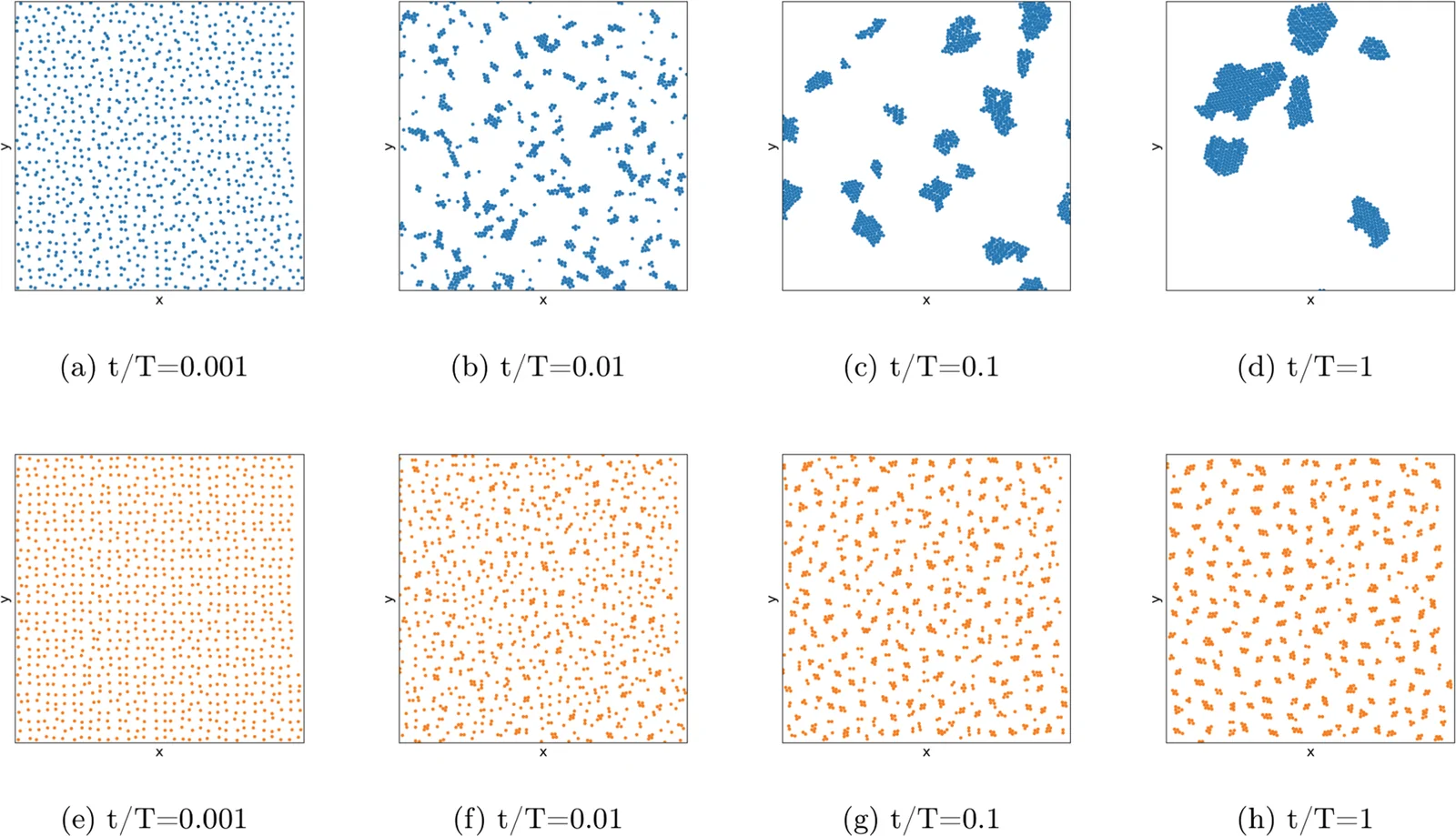
Emergent behavior of the microrobotic swarm using LJ interactions, periodic boundary conditions, and a lattice-with-jitter initialization. Spatial configurations are shown at selected early times and at the final time for Newtonian transport (top row) and viscoelastic fractional Kelvin–Voigt transport (bottom row). Both cases use the same base active-particle parameters; the viscoelastic case additionally contains the fractional memory term. Newtonian drag promotes localized aggregation and compact clustering, whereas viscoelastic drag yields a more discrete and spatially distributed swarm. For animations, see the Electronic Supplementary Material

This qualitative difference becomes more visually apparent in Fig. 3, where the final spatial states are compared directly. Without viscoelasticity, the swarm exhibits more pronounced clustering and localized accumulation. With viscoelasticity, the particles remain more spatially distributed, more discrete, and less prone to compact agglomeration. In other words, the Newtonian setting favors stronger collective packing, whereas the viscoelastic setting promotes a more separated and cluttered swarm morphology. From a targeted delivery viewpoint, the latter is appealing because excessive aggregation may reduce effective coverage, hinder penetration into confined biological spaces, and compromise controllability of the carrier population.

The clustering shown in the Newtonian snapshots is interpreted primarily as activity-driven, MIPS-like clustering rather than as a purely cohesive LJ aggregate. This distinction is important. For simulations using the WCA potential, the pair interaction is purely repulsive, so cluster formation cannot arise from an attractive tail. Instead, it originates from the ABP mechanism: persistent self-propulsion drives particles into dense regions, collisions and local crowding reduce their escape rate, and the resulting density-motility feedback promotes phase separation or MIPS-like aggregation. For simulations using the LJ potential, the attractive tail can further strengthen or modify the clusters, but it is not the only clustering mechanism. Therefore, we interpret the observed aggregation as primarily active-matter clustering, with LJ cohesion acting as an additional contribution only in those panels where LJ interactions are explicitly used.

Figure 3 also makes the time-dependent nature of the emergent behavior much clearer than a final-state comparison alone. In the Newtonian medium, clustering is not only present at the end of the simulation but already becomes clearly visible by t/T=0.1 in Fig. 3c, where the swarm has already reorganized into compact, spatially localized groups. By the time one reaches Fig. 3d, this organization has intensified further, and the system is dominated by pronounced aggregated clusters. The viscoelastic case follows a very different route. Even at the corresponding intermediate and final stages in Fig. 3g and h, the swarm remains much more scattered, with no comparable transition toward compact collective packing. This contrast is important because it shows that viscoelasticity does not merely change the final appearance of the swarm; it changes the entire temporal pathway by which structure emerges. From the delivery viewpoint, that is encouraging, since a swarm that stays distributed over time is more likely to preserve functional transport and avoid premature agglomeration.

**Fig. 4:**
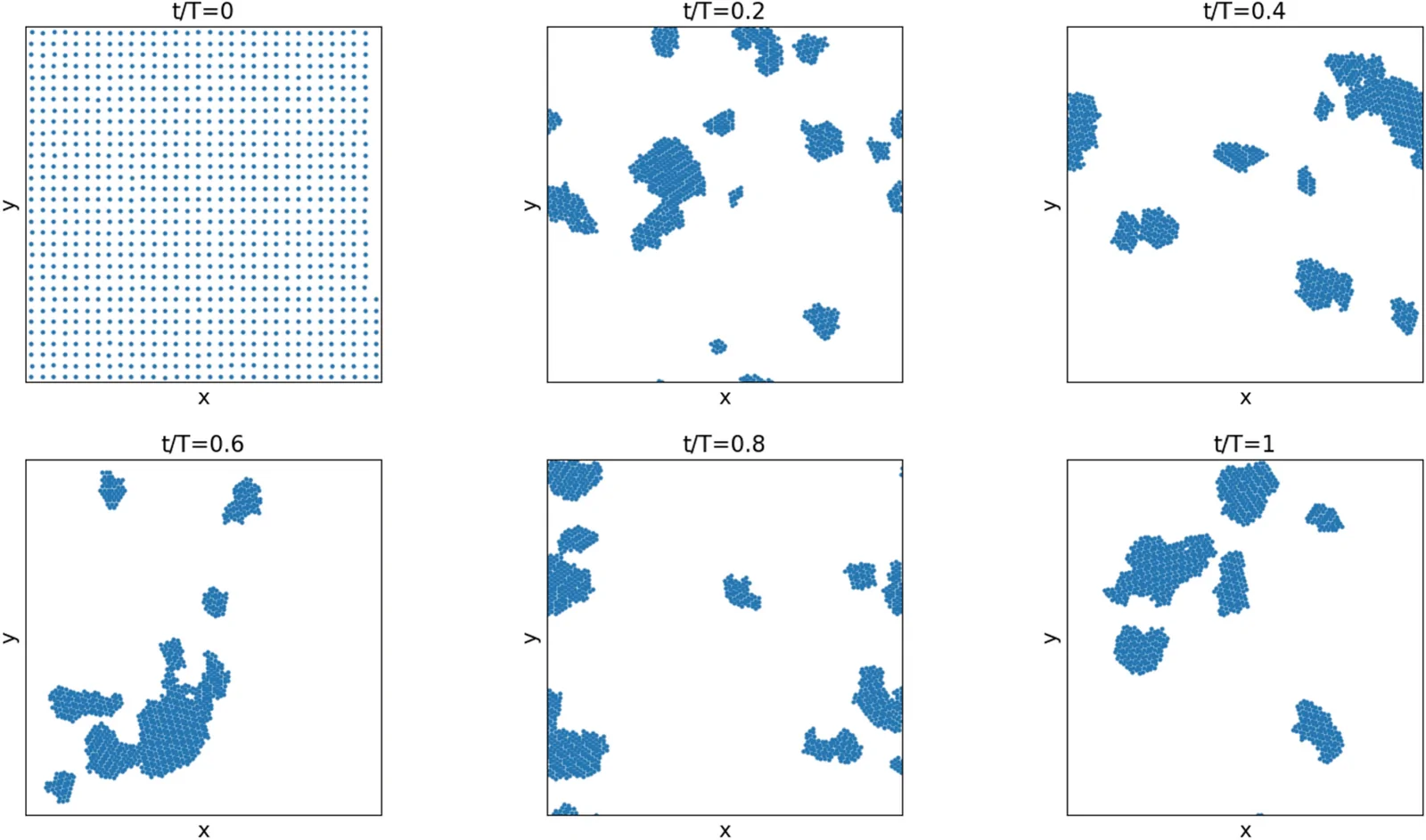
Expanded chronological view of the same Newtonian LJ simulation shown in the top row of Fig. 3, using periodic boundary conditions and a lattice-with-jitter initialization. The more closely spaced snapshots show the progressive development of localized clusters. For animations, see the Electronic Supplementary Material

**Fig. 5:**
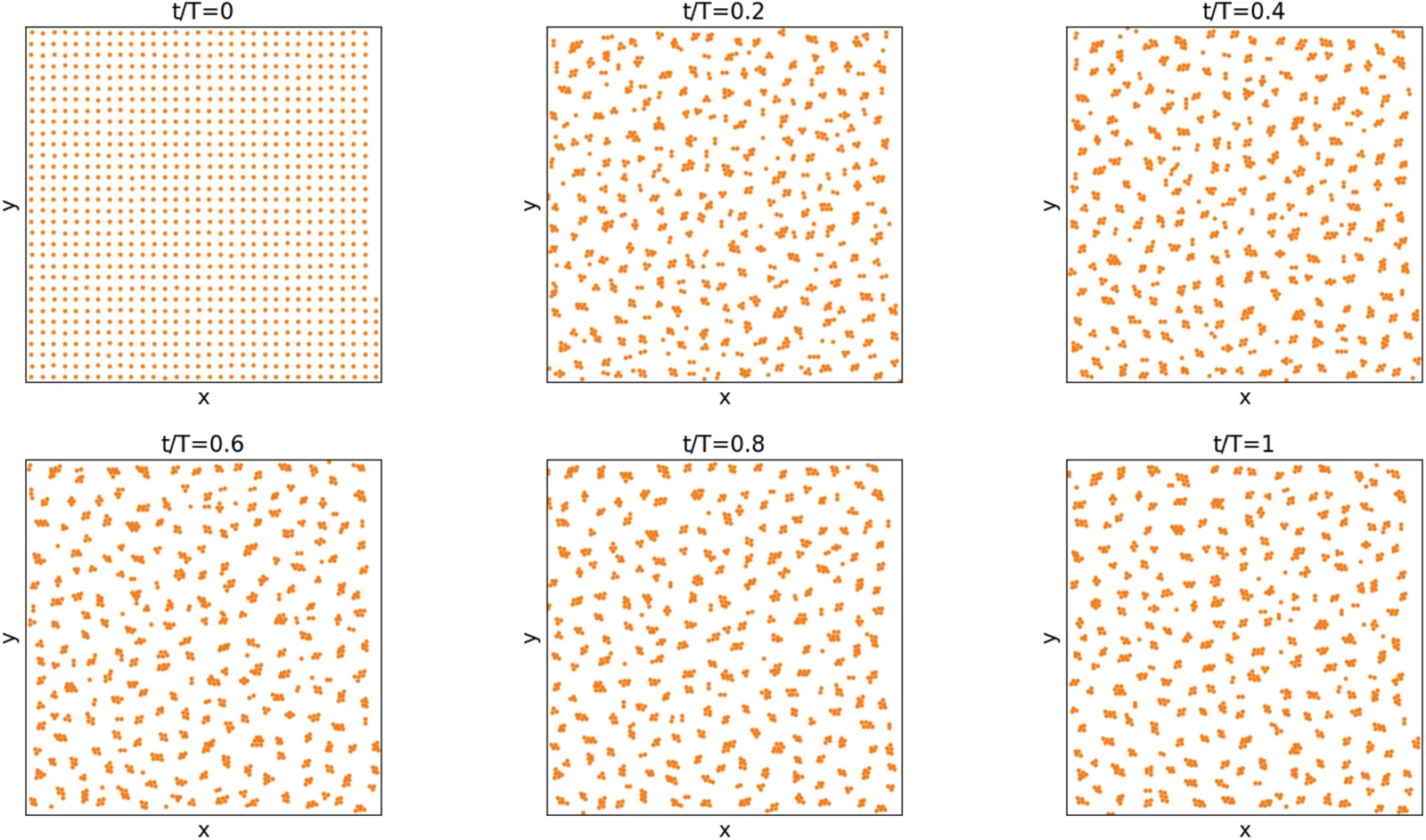
Expanded chronological view of the same viscoelastic fractional Kelvin–Voigt LJ simulation shown in the bottom row of Fig. 3, using periodic boundary conditions and a lattice-with-jitter initialization. The more closely spaced snapshots show that the swarm remains comparatively distributed throughout the observation window. For animations, see the Electronic Supplementary Material

Figures 4 and 5 provide expanded chronological views of the same Newtonian and viscoelastic simulations shown in Fig. 3, respectively. The more closely spaced snapshots reinforce the same interpretation. In the Newtonian case, localized clustering develops progressively over time, suggesting stronger cohesive accumulation and a higher likelihood of congestion or jamming-like organization. In the viscoelastic case, the swarm remains much more scattered throughout the observation window, with negligible large-scale clustering. This qualitative suppression of aggregation is the key message of the preliminary results. It suggests that the non-Newtonian and viscoelastic character of physiological media may, under suitable conditions, help maintain a transport-favorable distribution of microrobotic carriers rather than forcing them into overly cohesive assemblies.

**Fig. 6:**
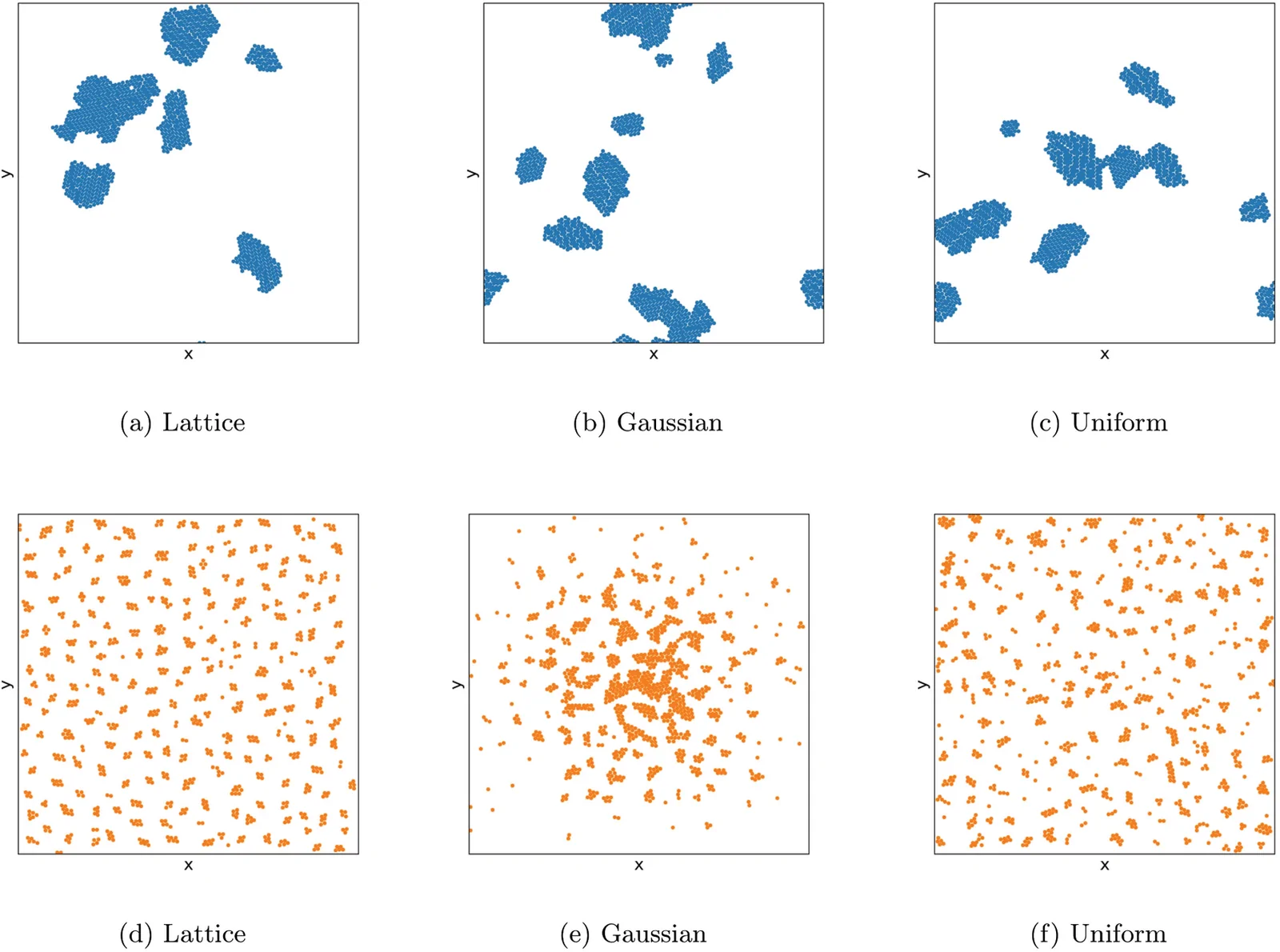
Final spatial configurations at t/T=1 of the microrobotic swarm using LJ interactions and periodic boundary conditions. Newtonian transport is shown in the top row and viscoelastic fractional Kelvin–Voigt transport in the bottom row for lattice, Gaussian, and uniform initial particle distributions. Newtonian drag produces compact localized clusters, whereas viscoelastic drag maintains a comparatively dispersed swarm morphology. For animations, see the Electronic Supplementary Material

**Fig. 7:**
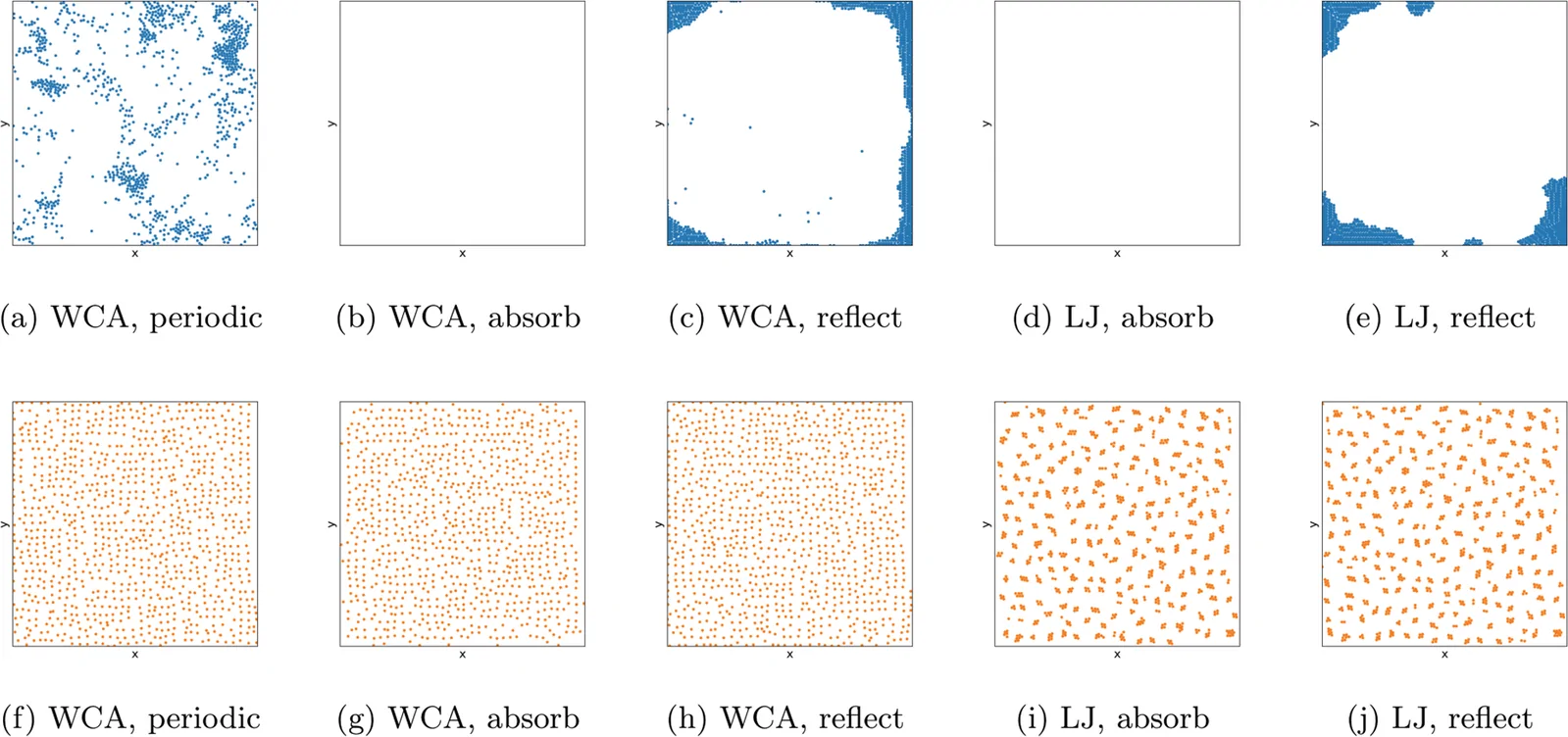
Final spatial configurations (at t/T=1) of the microrobotic swarm for Newtonian (top row) and viscoelastic media (bottom row) for different interaction potentials and boundary conditions. The sub-captions represent the interaction potential and boundary condition, respectively. Yet again, Newtonian drag drives localized particle aggregation into compact clusters, whereas viscoelastic drag induces a discrete, well-separated distribution that maintains a comparatively dispersed swarm morphology. For animations demonstrating the motion of the active Brownian particle microrobotic swarms, see the Electronic Supplementary Material

Figure 6 shows that this contrast is not an artifact of one specially chosen initialization. In the Newtonian medium, clustering remains the dominant outcome whether the particles are initialized on a lattice, in a Gaussian cloud, or from a uniform distribution; see Fig. 6a–c. The exact cluster shape changes, but the broad tendency toward localized aggregation survives all three initial conditions. In the viscoelastic case, however, the final states in Fig. 6d–f remain far more dispersed. The lattice and uniform initializations show almost no meaningful clustering by the final time, while the Gaussian initialization still exhibits only a comparatively mild residual central accumulation. This robustness across initial conditions strengthens the main interpretation of the paper: the aggregation tendency is primarily controlled by the constitutive character of the surrounding medium rather than by one accidental starting arrangement. That makes the viscoelastic effect much more compelling from a transport perspective, because it suggests a genuinely stabilizing mechanism against unwanted agglomeration.

Figure 7a extends the same logic to interaction potentials and boundary conditions. For practical applications, if the microrobots are very small compared with the surrounding region of interest and wall effects are negligible, then the periodic case is often the natural idealization. In that setting, Fig. 7a (a, f) shows that even the purely repulsive WCA case still produces pronounced clustering in the Newtonian medium, much like the LJ-based cases discussed earlier. The absorbing case in Fig. 7a (b, d, g, i) tells a different but equally useful logic: by the end of the simulation, the particles have effectively left the domain of interest, which in a transport reading may be interpreted as loss from the relevant target region. The reflecting case in Fig. 7a (c, e, h, j) reveals strong boundary accumulation, meaning that particles are redirected and eventually pile up near the confining walls. What is especially striking, however, is that across these different potentials and boundary scenarios, the same broad constitutive contrast remains intact. Newtonian transport continues to favor aggregation, clustering, or accumulation, whereas viscoelastic transport suppresses compact clustering much more effectively. This persistence of the main trend across modeling choices makes the result particularly exciting: the beneficial anti-aggregation role of viscoelasticity is not fragile but appears consistently across different simulation conditions.

Taken together, these figures support our main biomedical narrative: when one compares Newtonian and viscoelastic transport at the level of collective swarm morphology, viscoelasticity appears to act as a mechanically beneficial ingredient that limits excessive aggregation. This is encouraging for microrobotic drug delivery, because a more spatially distributed carrier population is, in principle, better suited for coverage, controllable transport, and payload dispersion across complex bodily environments. The present results are, therefore, best understood as a qualitative proof-of-concept and a perspective-level design signal, motivating future studies with broader parameter exploration, richer transport metrics, and application-specific geometries.

## Discussion and outlook

The existing scholarship discussed earlier changes how the model should be perceived. If one looks only at the equations, the framework appears to be a standard ABP simulation with interactions and memory. However, the broader literature shows that the same mathematical ingredients have different meanings in different physical settings. For instance, in Vicsek-type models, alignment is often interpreted as local heading consensus or behavioral imitation [40]. In colloidal and bacterial systems, apparent alignment may also arise from hydrodynamic coupling, steric collisions, external fields, or boundary-mediated effects [25, 53, 55]. Therefore, $K_{\textrm{align}}$ should be interpreted cautiously. In a robotic swarm, it may represent a controller gain; in a microrobotic colloid, it may represent an effective outcome of field coupling; and in a biological suspension, it may summarize unresolved flow-orientation coupling.

The LJ/WCA comparison is also central to the interpretation of clustering. The LJ potential contains an attractive tail and can therefore introduce explicit cohesion, whereas the WCA potential retains only the repulsive branch of the interaction [35, 36]. This distinction helps avoid the common mistake of calling every dense state an attraction-driven aggregate. In ABPs, clustering and phase separation can also arise from activity, persistence, steric slowing, and the density-motility feedback associated with MIPS-like behavior [37–39]. The fractional Kelvin–Voigt term adds a different physical ingredient: it treats the medium as a source of history-dependent drag. Fish-school hydrodynamics and fluid-stigmergy studies show that the medium can also transmit information through wakes, vortices, and flow-mediated interactions [68, 69, 72]. In biomedical gels, mucus, blood, and extracellular matrix, both views may matter: the medium resists motion and may also guide, delay, or disrupt collective organization.

The neural-network study on commanding emergent behavior demonstrates that inverse design of interaction rules is becoming realistic [73]. However, a learned rule must still obey the mechanical constraints of drag, confinement, actuation limits, collision, and safety. The present model can, therefore, serve as a physics layer beneath learning-based control: first identify what mechanical regimes are feasible, then use optimization or learning to tune within that feasible set. Finally, the cross-scale catheter-swarm thrombus study shows that a biomedical swarm cannot be judged only by how well it reaches a target [7]. One must also ask whether it can be localized, whether it can perform mechanical work, whether debris can be controlled, whether residual particles are safe, and whether the whole system can be retrieved. This broadens the meaning of *success* in a simulation. A high clustering fraction or high transport speed is not enough unless it maps to a clinically useful and controllable task.

From a biomedical viewpoint, the present framework can be read as a mechanics-first model for how active drug carriers or microrobotic swarms move, cluster, spread, and retain cargo in physiologically complex media. That is directly relevant to targeted transport through blood and microvascular networks, localization near tumor interiors, brain-directed delivery where blood-brain barrier constraints and confined tissue spaces matter, and access to deep-seated lesions or poorly perfused wound beds where passive delivery is limited. The central modeling ingredients of this paper — low-Re drag, short-range interaction, alignment, and memory — map naturally onto questions of medical navigation, local retention, cooperative coverage, and cargo concentration *in vivo*.

The principal limitation is the absence of resolved hydrodynamic interactions. The model uses drag plus pair potentials, not a full fluid flow or mobility tensor. This is appropriate for a transparent overdamped baseline, but long-range hydrodynamic interactions between moving particles can matter substantially in bacterial suspensions, fish schools, and some microswimmer systems [12, 29, 53, 69]. A future modeling strategy could replace the local drag law with a mobility operator, Stokesian dynamics approximation, immersed boundary formulation, or data-driven hydrodynamic closure. A second limitation is noise modeling in the viscoelastic setting. The fractional Kelvin–Voigt extension modifies drag but retains white noise for practical simplicity. A fully thermodynamically consistent viscoelastic bath would require a generalized Langevin formulation with memory-consistent colored noise [74]. The present choice is intentionally simpler because the immediate goal is to isolate the effect of history-dependent resistance on collective pattern formation.

A third limitation is all-pairs computational scaling. Dense vectorization has $\mathcal {O}(N^2)$ cost per step. It is excellent for transparent reference runs and moderate *N*, but not asymptotically optimal. Neighbor-list acceleration is the next obvious computational improvement. A fourth limitation is biological and clinical realism. If the goal were patient-specific prediction or device optimization for a particular intervention, additional ingredients would eventually be needed: vessel walls and endothelium, adhesion, red-blood-cell crowding, immune interactions, deformable carriers, porous tissue infiltration, biochemical binding, imaging feedback, and realistic geometry extracted from medical images. The present computational model should, therefore, be viewed as a baseline mechanics layer for richer and more advanced application-specific models.

## Conclusions

This work reviews the foundational milestones and current status of microrobotic swarms, evaluating their prospective biomedical applications and predictive modeling frameworks to delineate a future research trajectory. Consequently, this synthesis offers significant value to investigators aiming to propel this dynamic discipline forward. By contextualizing seminal contributions from recent decades and integrating the existing literature, we demonstrate the utility of this minimal computational architecture and its strategic roadmap. For example, studies of bacterial living fluids emphasize hydrodynamic stress-orientation coupling. Field-driven colloidal and microrobotic swarms demonstrate that external actuation can create programmable collective states. Fish-school hydrodynamics shows that spatial pattern and phase can determine energetic cost and benefit. Neural-network control suggests that interaction rules can be inversely designed to produce target morphologies. Cross-scale biomedical swarm work implies that delivery, disruption, retrieval, and safety must eventually be treated together.

Furthermore, we have presented an explicit route from mechanics to an algorithm for active many-particle dynamics in low-Reynolds-number and viscoelastic settings. Starting from Newton’s law, we introduced drag and stochastic forcing to obtain the underdamped Langevin equation. We then passed on to the overdamped limit appropriate to microscale agents in strongly dissipative media. We constructed an active interacting swarm model with self-propulsion, local alignment, and short-range pair interactions of LJ or WCA type. We then extended the Newtonian-drag law to a fractional Kelvin–Voigt form to represent gel-like memory. This extension introduces a physically interpretable history-dependent resistance. To connect theory with practice, we mapped the resulting stochastic dynamics to an explicit algorithm based on Euler–Maruyama time integration, minimum-image periodic distances, and truncated Grünwald-Letnikov history convolution.

We completely agree that the proposed computational approach is not the most elaborate. However, its value lies in clarity, reproducibility, and extensibility, given that this study lays the perspective and prospects. Therefore, it provides a clean baseline from which one can move in several directions: larger systems via neighbor lists, richer environments via generalized Langevin or fluid-coupled descriptions, more realistic interactions via anisotropy or sensing rules, and more refined diagnostics via correlation functions and structure factors. In that sense, the present paper is both a standalone modeling framework and a launch point for more specialized studies of collective dynamics in complex soft environments.

From the drug-delivery viewpoint, the central message is especially important: for many biomedical applications, aggregation is not automatically desirable. If the transported microparticles or microrobots form overly cohesive clusters, jam in confined spaces, or collapse into dense flocks, their effective coverage, controllability, penetration, and payload release can all be compromised. The present simulations, therefore, suggest a practically useful distinction between two transport scenarios. Under Newtonian drag, the particles show a stronger tendency toward clustering, aggregation, and collective congestion. Under viscoelastic Kelvin–Voigt-type drag, by contrast, they can remain more separated, more discrete, and less prone to irreversible agglomeration. In the context of targeted delivery, this latter behavior is attractive because it can preserve functional dispersion while still allowing coordinated transport. The additional comparisons across time, initialization, interaction potential, and boundary condition further suggest that this trend is not tied to one isolated setup but reflects a broader mechanical tendency of viscoelastic transport to resist compact collective collapse.

Equally important, the results indicate that the swarm response is genuinely emergent and time-dependent rather than a trivial relaxation toward a single static state. This is visible in the nontrivial temporal behavior of polarization and in the evolving spatial morphologies across initial conditions, boundaries, and time horizons. Such behavior is relevant because therapeutic transport and payload release in the body also occur over finite time windows: transport to target regions may occur on short or intermediate physiological timescales depending on route and carrier design, while release itself may be immediate, delayed, or sustained. For that reason, a transient collective organization may be just as important as an eventual long-term structure. Viewed in this way, our results provide an encouraging signal: the non-Newtonian and viscoelastic character of bodily media may help reduce harmful aggregation and thereby improve the prospects for effective microrobotic drug delivery. We, therefore, hope that this computational perspective will motivate future studies that connect swarm mechanics more directly to delivery efficiency, target retention, release kinetics, and clinical translation.

## Supplementary Information

Below is the link to the electronic supplementary material.

Supplementary file 1 (mp4 13165 KB)

Supplementary file 2 (mp4 13266 KB)

Supplementary file 3 (mp4 13165 KB)

Supplementary file 4 (mp4 13187 KB)

Supplementary file 5 (mp4 13285 KB)

Supplementary file 6 (mp4 12774 KB)

Supplementary file 7 (mp4 13007 KB)

Supplementary file 8 (mp4 13011 KB)

Supplementary file 9 (mp4 13022 KB)

Supplementary file 10 (mp4 13075 KB)

Supplementary file 11 (mp4 4288 KB)

Supplementary file 12 (mp4 12917 KB)

Supplementary file 13 (mp4 13087 KB)

Supplementary file 14 (mp4 6768 KB)

Supplementary file 15 (mp4 13332 KB)

Supplementary file 16 (mp4 12779 KB)

Supplementary file 17 (pdf 88 KB)

## Data Availability

No new experimental data were generated in this perspective/framework article. Simulation data can be generated from the documented model and are available upon reasonable request.

## List of symbols

*N*: Number of particles in the swarm
*d*: Spatial dimension of the simulation domain
Ω: Computational domain
*L*: Linear size of the computational domain
*t*: Time
Δt: Numerical time-step size
*T*: Final simulation time or observation time
$\textbf{r}_i(t)$: Position vector of particle *i* at time *t*
$\textbf{v}_i(t)=\dot{\textbf{r}}_i(t)$: Velocity vector of particle *i*
$\theta _i(t)$: Heading angle of particle *i* in two dimensions
$\textbf{n}_i(t)$: Unit heading or propulsion direction of particle *i*
*m*: Particle mass in the underdamped Langevin description
$\textbf{F}_i$: Total deterministic force acting on particle *i*
$\textbf{F}_{ij}$: Pair force exerted on particle *i* by particle *j*
*U*(*r*): Pair-interaction potential as a function of interparticle distance *r*
ζ: Translational drag coefficient
$$\mu =1/\zeta $$: Mobility
$k_BT$: Thermal or effective stochastic energy scale
$D_t$: Translational diffusion coefficient
$D_r$: Rotational diffusion coefficient
$\textbf{W}_i(t)$: Wiener process associated with translational Brownian motion
$\boldsymbol{\xi }_i^n$: Standard Gaussian random vector used in the discrete time update
$\boldsymbol{\eta }_i(t)$: Gaussian white-noise process
$v_0$: Self-propulsion speed
$R_{\textrm{align}}$: Metric alignment radius
$K_{\textrm{align}}$: Alignment rate or alignment strength
ε: Pair-interaction energy scale in the LJ and WCA potentials
σ: Effective particle diameter or interaction length scale
$r_c$: Interaction cutoff radius
$r_{\min }$: Minimum pair-distance regularization used for numerical stabilization
$F_{\max }$: Maximum force magnitude used for force clipping
α: Fractional order of the viscoelastic memory term
$\zeta _\alpha$: Fractional-memory drag coefficient
^C^$D_t^\alpha$: Caputo fractional derivative of order α
$\mathcal {D}_\alpha$: Discrete truncated-history approximation of ^C^$D_t^\alpha$
$K_\alpha (t-s)$: Power-law memory kernel associated with the Caputo derivative
*M*: Number of stored history terms in the truncated fractional convolution
$T_{\textrm{mem}}=M\Delta t$: Effective memory window in the fractional derivative approximation
*E*: Elastic modulus in the Kelvin-Voigt description
η: Viscosity in the classical Kelvin-Voigt model
$\eta _\alpha$: Fractional viscosity-like coefficient in the fractional Kelvin-Voigt model
ε(t): Strain in the Kelvin-Voigt description
*P*: Probability density in the Fokker-Planck description

## Abbreviation

**Abbreviation**: **Meaning**
ABP: Active Brownian particle
LJ: Lennard-Jones
WCA: Weeks-Chandler-Andersen

## Code Availability Statement

Codes are available upon reasonable request.
