# Supplementary material for: Emergent collective dynamics of microrobotic swarms in viscoelastic media: a computational perspective
Source: Eur Phys J E Soft Matter. 2026 Aug 28;49(9):80. doi: 10.1140/epje/s10189-026-00617-4 (PMC13525069; doi:10.1140/epje/s10189-026-00617-4)
Supplement: Supplementary file 1 [file 10189_2026_617_MOESM17_ESM.pdf]

# Supplementary Material

The filenames are representative of the following components:

## Fluid & Medium Types

- **Newtonian**: a Newtonian fluid that is purely viscous in nature
- **Frac_kv**: a fractional Kelvin-Voigt medium that is viscoelastic in nature

## Interaction Potentials

- **lj**: Lennard-Jones potential
- **wca**: Weeks-Chandler-Andersen potential

## Initial Particle Distributions

- **uniform**: uniformly distributed particles at the start of simulation
- **gaussian**: gaussian distribution of particles at the start of simulation

## Boundary Conditions

- **periodic**: periodic boundary condition in x- and y-axes
- **reflect**: boundaries reflect the particles and the particles do not leave the region
- **absorb**: particles are not considered further (lost) once they are out of the simulation region
